# Profiling of human lymphocytes reveals a specific network of protein kinases modulated by endurance training status

**DOI:** 10.1038/s41598-020-57676-6

**Published:** 2020-01-21

**Authors:** Katharina Alack, Astrid Weiss, Karsten Krüger, Mona Höret, Ralph Schermuly, Torsten Frech, Martin Eggert, Frank-Christoph Mooren

**Affiliations:** 1 Department of Exercise Physiology and Sports Therapy, Institute of Sports Sciences, Justus-Liebig-University, Giessen, Germany; 2 Member of the German Center for Lung Research (DZL), Cardio-Pulmonary Institute (CPI), Justus-Liebig-University, Giessen, Germany; 3 Center for Extracorporeal Organ Support, Department of Internal Medicine, Universitätsmedizin Rostock, Rostock, Germany; 4 Witten/Herdecke University, Faculty of Health/School of Medicine, Witten, Germany

**Keywords:** Cellular immunity, Lymphocytes

## Abstract

To date, the effects of endurance exercise training on lymphocyte physiology at the kinome level are largely unknown. Therefore, the present study used a highly sensitive peptide-based kinase activity profiling approach to investigate if the basal activity of tyrosine (Tyr) and serine/threonine (Ser/Thr) kinases of human lymphocytes is affected by the aerobic endurance training status. Results revealed that the activity of various tyrosine kinases of the FGFR family and ZAP70 was increased, whereas the activity of multiple Ser/Thr kinases such as IKK_α_, CaMK4, PKA_α_, PKC_α+δ_ (among others) was decreased in lymphocytes of endurance trained athletes (ET). Moreover, functional associations between several differentially regulated kinases in ET-derived lymphocytes were demonstrated by phylogenetic mapping and network analysis. Especially, Ser/Thr kinases of the AGC-kinase (protein kinase A, G, and C) family represent exercise-sensitive key components within the lymphocytes kinase network that may mediate the long-term effects of endurance training. Furthermore, KEGG (Kyoto Encyclopedia of Genes and Genomes) and Reactome pathway analysis indicate that Ras as well as intracellular signaling by second messengers were found to be enriched in the ET individuals. Overall, our data suggest that endurance exercise training improves the adaptive immune competence by modulating the activity of multiple protein kinases in human lymphocytes.

## Introduction

Aerobic endurance exercise induces specific physiological demands which require the regulation of diverse signaling pathways for rapid cellular adaptation. On this occasion, phosphorylation-regulated signaling pathways play an essential role in enabling cells to respond quickly and adequate to various exercise-associated cellular stressors such as hypoxia, reactive oxygen species, and alterations in calcium homeostasis^1–4^. Protein kinases represent an important group of intracellular enzymes that play a crucial role in signal transduction cascades, including those involved in cell growth control, cell proliferation, and regulation of immunological responses^5^. More precisely, protein kinases phosphorylate their cellular target proteins by attaching the gamma-phosphoryl group of adenosine triphosphate (ATP) to the side chains of tyrosine, serine or threonine residues^5,6^. Thus, protein phosphorylation, mediated by protein kinases, displays an essential and indispensable post-translational control mechanism in cellular signal transduction^5,7^.

The investigation of phospho-proteomic adaptations to exercise is a very recent field of research and there are just a few of pilot studies to date that focus on exercise-induced changes in skeletal or heart muscle^8–11^. Global pospho-proteomic analysis in human skeletal muscle biopsies from untrained healthy male subjects before and after a single bout of high-intensity cycle exercise revealed 1,004 exercise-regulated phospho-sites on 562 proteins^9^. In detail, substrates of several prominent kinases such as AMPK, PKA, CaMK and MAPK have been modulated by exercise-induced signaling cascades^9^. So far, the effect of exercise training on the kinase activity in lymphocytes has been analyzed only under pathophysiological conditions for individual selected candidates^12,13^.

Indeed, aerobic endurance training is well-known as a solid therapeutic option in the prevention and treatment of several metabolic, immunological and autoimmune diseases^14,15^. Protein kinases represent a promising novel target for pharmacological interventions as the dysfunction of protein kinases plays a major role in the pathogenesis of numerous diseases (e.g. cancer, pulmonary and cardiovascular diseases)^16–19^. Actually, specific kinase inhibitors are used successfully in cancer therapy^20,21^. Therefore, identifying exercise-induced adaptations in protein kinase activity may be a promising approach for the identification of new pharmacological drug targets mimicking the health benefits of exercise training^9^. Furthermore, the identification of protein kinases modulated by exercise training is particularly of great interest for a potential future use of kinases as biomarkers for monitoring exercise and training responses.

As part of the adaptive immune response, the main focus of lymphocytes is the targeted defense against foreign substances, especially infectious agents^22^. Yet, there is broad evidence that exercise influences the regulation of various lymphocyte functions such as differentiation, migration or apoptosis^23–25^. Moreover, molecular genetic adaptations as reaction to endurance exercise training in human lymphocytes were already demonstrated in a recent study by our group^26^.

However, it has not yet been investigated whether endurance exercise training can induce long-term adaptations in the activity of multiple tyrosine and serine/threonine kinases in human lymphocytes. To date, it is largely unknown how phosphorylation-regulated signaling pathways are influenced by the exercise-induced modulation of the protein kinase activity in immune cells of the peripheral blood. Therefore, the major interest of the present study is to define key components within the kinase network of human lymphocytes by a peptide-based kinase activity assay that provide the long-term effects of endurance exercise training.

## Results

### Intensity of peptide substrate phosphorylation of Tyr and Ser/Thr kinase microarray differs between endurance-trained athletes (ET) and untrained subjects (UT)

**Figure 1:**
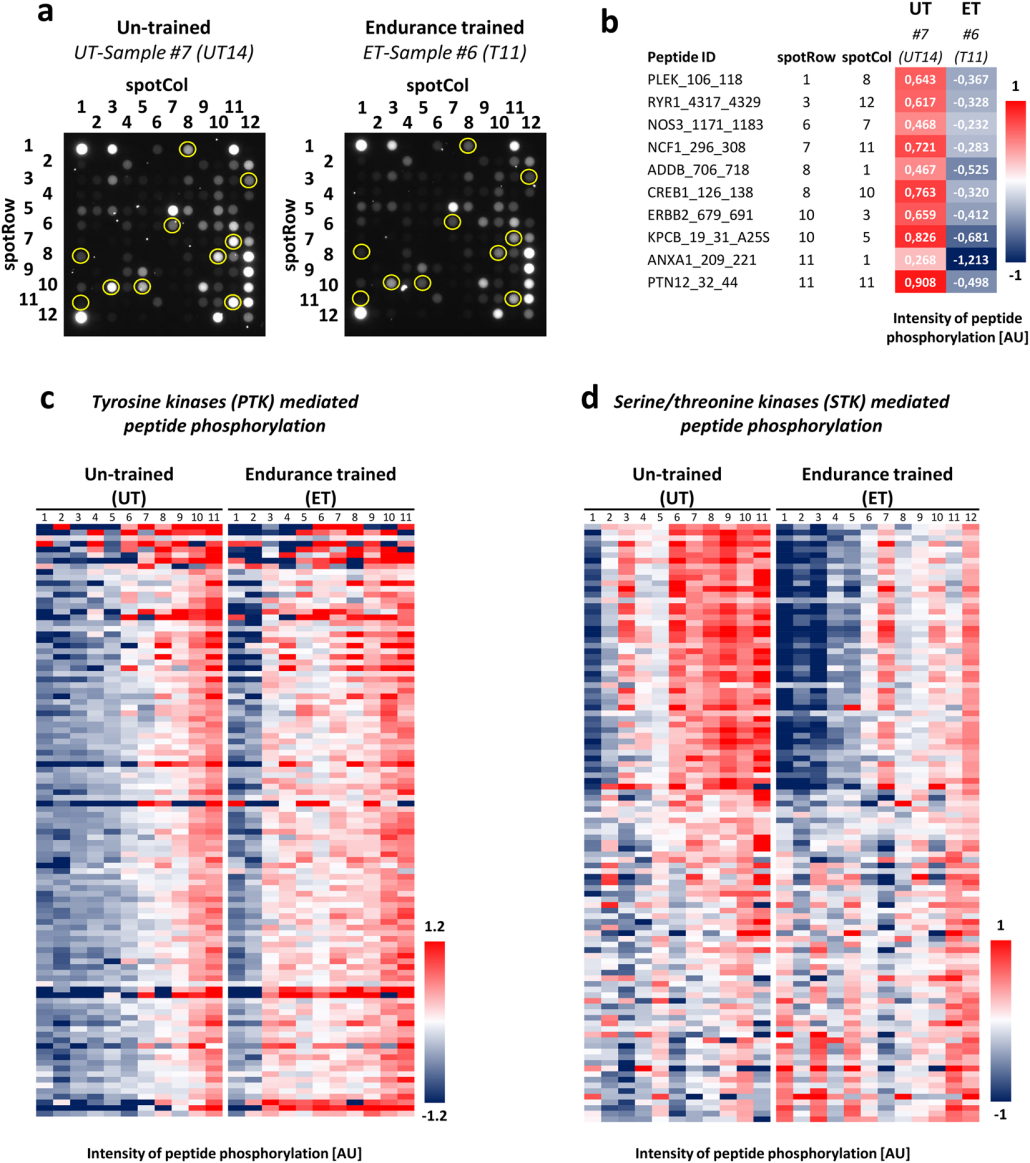
Signatures of peptide phosphorylation due to differential kinase activity in lymphocytes isolated from untrained (UT) and endurance-trained (ET) participants. (**a**) Raw data images are shown for one representative sample from both study groups obtained during a STK run, i.e. UT #7 and ET #6, with highlighted signals for distinct peptides which display a clear change in phosphorylation (yellow mark-ups). (**b**) Peptide identities (ID) for the marked-up peptides in Fig. 1a are given with their respective position on the STK chip as well as their numeric value of phosphorylation intensity after log-transformation and normalization by date centering. (**c**) The heat map visualizes the individual intensity of phosphorylation of all quality controlled peptides on PTK chips which serve as substrates for tyrosine kinases. All lymphocyte samples from UT and ET subjects are shown. (**d**) The heat map visualizes the individual intensity of phosphorylation of all quality controlled peptides on STK chips which serve as substrates for serine/threonine kinases. All lymphocyte samples from UT and ET subjects are shown. AU: Arbitrary units.

Equal amounts of lymphocyte lysate from ET and UT participants were analyzed for their tyrosine (Tyr) as well as serine/threonine (Ser/Thr) kinase activity on peptide microarray PTK (for Tyr kinases) and STK chips (for Ser/Thr kinases). A representative illustration of raw data images obtained during an STK run is depicted in Fig. 1a. Here, a single sample from each experimental group, i.e. untrained versus endurance-trained, is shown with highlighted spots of some of the most deregulated phospho-peptides. Densitometric quantification of those marked phospho-peptides was performed, followed by log-transformation and data normalization by date centering resulting in a graphical heat map (Fig. 1b). A complete presentation of the differential pattern of peptide phosphorylation is also given in corresponding heat maps for all substrates of tyrosine-kinases (PTK, Fig. 1c) and serine/threonine kinases (STK, Fig. 1d). The underlying numerical values of the phosphorylation intensity of the peptide substrates spotted on the Tyr kinase microarray are depicted in Table S1. Predominantly, the intensity of tyrosine substrate peptide phosphorylation was markedly increased for the most peptides in ET-derived lymphocytes (e.g. VGFR2_1168_1180 and ZAP70_485_497). In contrast, the intensity of phosphorylation was lower for the most part of Ser/Thr peptide substrates (e.g. CDN1A_139_151 and PTK6_436_448) in the group of athletes (Table S2). The data also display some interindividual variation for phosphorylation intensities (e.g. Ser/Thr array: UT9, ET9; Tyr array: ET6).

### Increase of Tyr kinase activity in ET-derived lymphocytes

**Figure 2:**
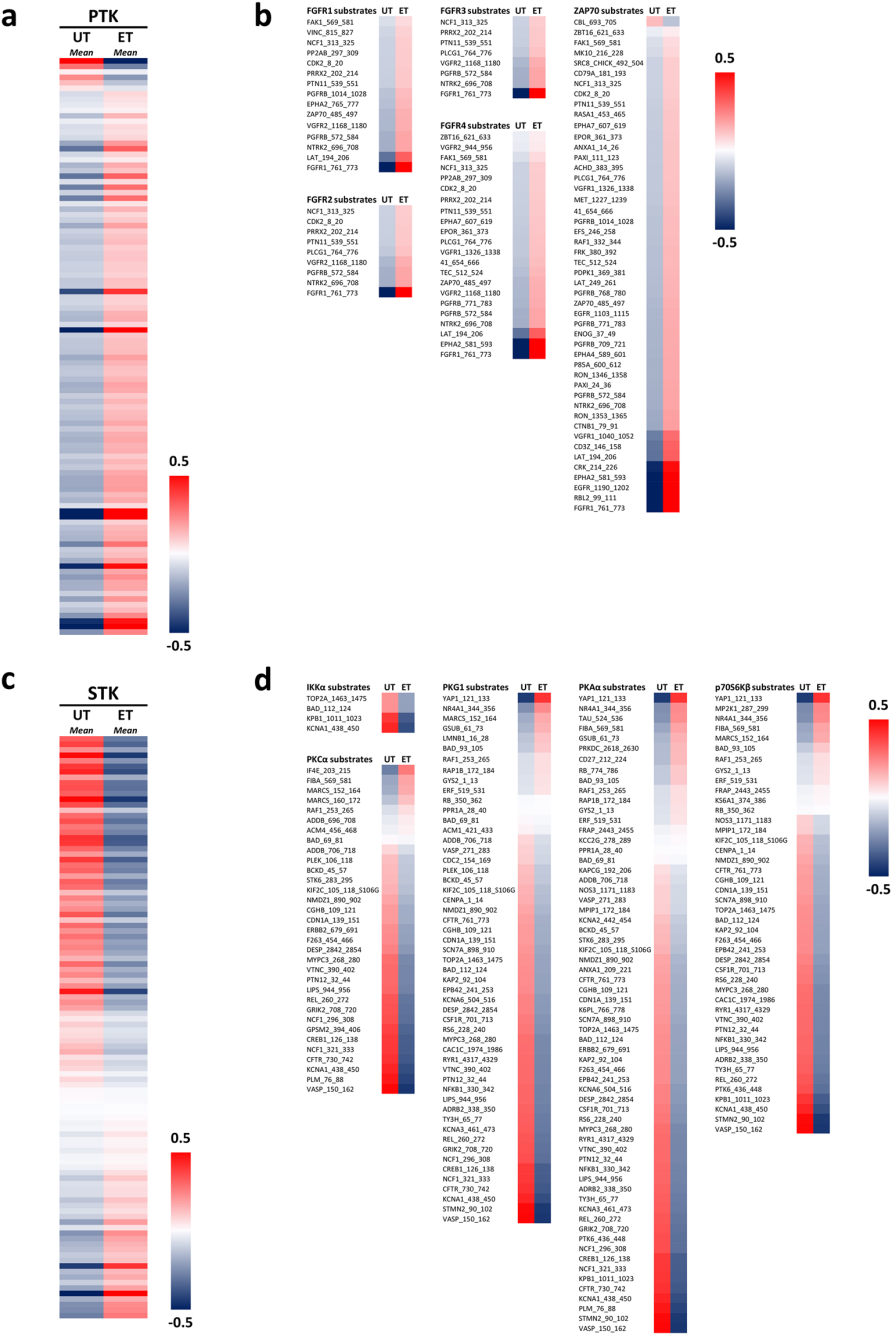
Heat maps of mathematical mean (average) peptide phosphorylation in lymphocytes from untrained (UT) and endurance-trained (ET) participants. (**a**) The heat map visualizes the mean phosphorylation of all quality controlled peptides on PTK chips which serve as substrates for all tyrosine kinases. (**b**) Selections of peptides (out of the complete list of phosphorylable peptides displayed in Fig. 2a) which were used to separately predict the increased upstream kinase activity for FGFR1, FGFR2, FGFR3, FGFR4, and ZAP70 in lymphocytes from ET subjects. (**c**) The heat map visualizes the mean phosphorylation of all quality controlled peptides on STK chips which serve as substrates for all serine/threonine kinases. (**d**) Selections of peptides (out of the complete list of phosphorylable peptides displayed in Fig. 2c) which were used to separately predict the decreased upstream kinase activity for IKKα, PKCα, PKG1, PKAα, and p70s6Kβ in lymphocytes from ET subjects. AU: Arbitrary units.

**Figure 3:**
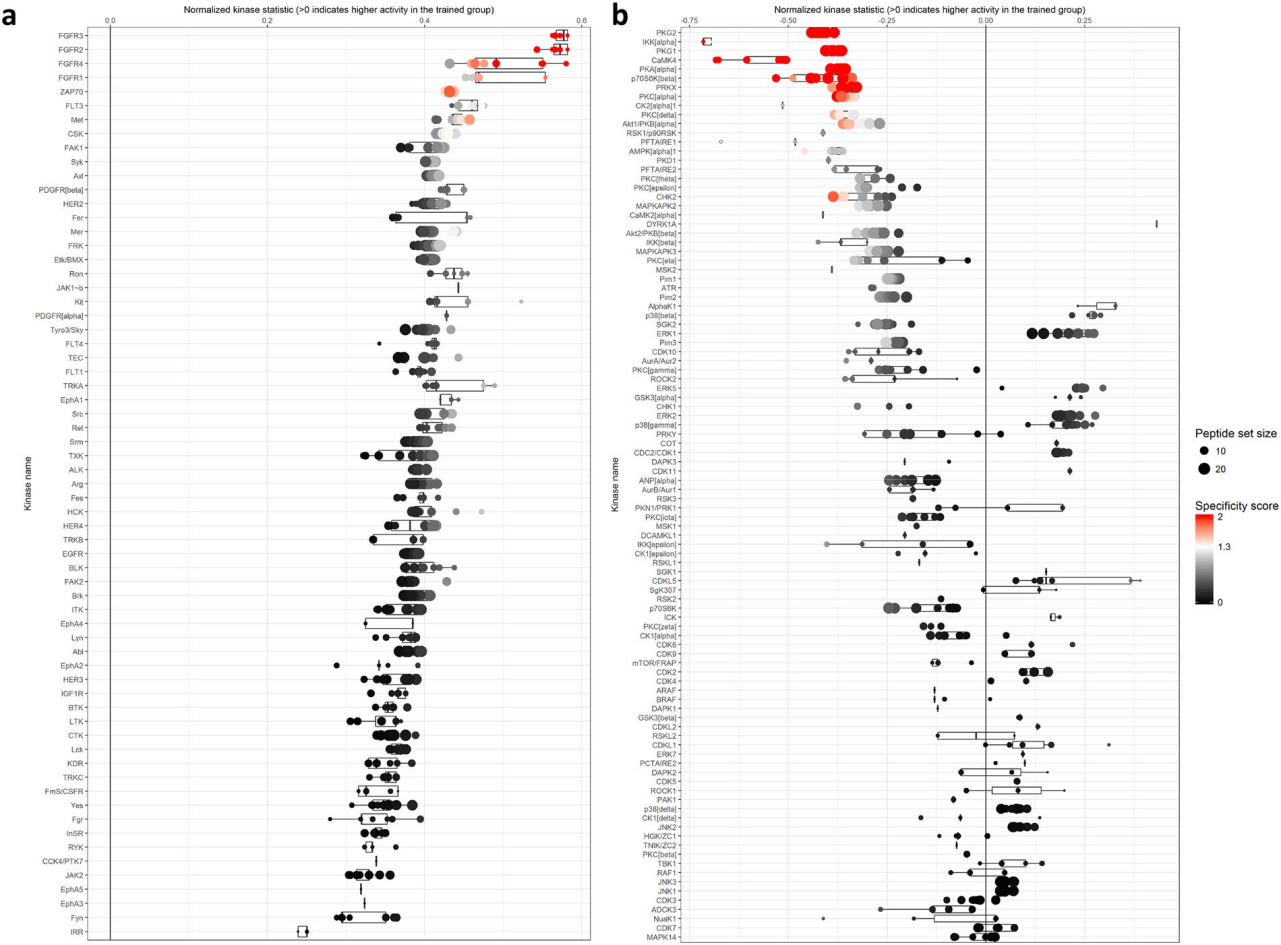
Median Final Score Plots of the tyrosine and serine/threonine kinase activity profiling in human lymphocytes of endurance trained athletes and untrained subjects. (**a**) Tyrosine kinase activity profiling: Median Final Score Plot. This plot shows putative upstream Tyrosine (Tyr) kinases ranked by their Final Score (Q). The top ranked Tyr kinases (FGFR3, FGFR2, FGFR4, FGFR1 and ZAP70) are more active in ET. In particular, the x-axis indicates the values for the normalized kinase statistic (s) (e.g. positive value (s > 0) = activity of the corresponding Tyr kinase is increased in ET). Each point of the plot represents the outcome of an individual analysis with a varying rank-cut off for adding upstream kinases for peptides. The size of each dot depicts the size of the peptide set which was used for the corresponding analysis. The specificity score (Qsp) is indicated by the color of the points. Q_sp_ logarithmic values > 1.3 (white [Q_sp_ = 1.3] to red color [Q_sp_ ≥ 2]) were considered as statistically relevant. FGFR: Fibroblast growth factor receptor; ZAP70: Zeta-chain-associated protein kinase 70; ET: endurance trained athletes; UT: untrained subjects. (**b**) Serine-/Threonine kinase activity profiling: Median Final Score Plot. This plot shows putative upstream Serine/Threonie (Ser/Thr) kinases ranked by their Final Score (Q). The top ranked Ser/Thr kinases (e.g. PKG2, IKKα, and PKG1) are less active in ET. In particular, the x-axis indicates the values for the normalized kinase statistic (s) (e.g. negative value [s < 0] = activity of the corresponding Ser/Thr kinase is decreased in ET). Each point of the plot represents the outcome of an individual analysis with a varying rank-cut off for adding upstream kinases for peptides. The size of each dot depicts the size of the peptide set which was used for the corresponding analysis. The specificity score (Q_sp_) is indicated by the color of the points. Q_sp_ logarithmic values > 1.3 (white [Q_sp_ = 1.3] to red color [Q_sp_ ≥ 2]) were considered as statistically relevant. PKG2: cGMP-depentent protein kinase 2; IKKα: Inhibitor of Nuclear Factor Kappa a Kinase Subunit alpha; PKG1: cGMP-depentent protein kinase 1; ET: endurance trained athletes; UT: untrained subjects.

Based on the detailed pattern of differential peptide phosphorylation for all individual samples (Fig. 1c) the mathematical mean of peptide phosphorylation including all PTK substrates for both experimental groups was calculated (Fig. 2a). The specific profiles for the distinct substrates used to predict an enhanced kinase activity for the top-five ranked candidates in ET-derived lymphocytes are reflected by the heat maps shown in Fig. 2b. The final ranking of the Tyr kinases based on median final score (Q) is presented in Fig. 3a. Additionally, the kinase statistic of the top 10 ranked Tyr kinases is shown in Table 1. Kinase profiling revealed a statistically relevant up-regulation of the activity of five Tyr kinases in the ET group (specificity score (Q_sp_) > 1.3). More precisely, four members of the fibroblast growth factor receptor family (FGFR3, FGFR2, FGFR4 and FGFR1) and the Zeta-chain-associated protein kinase 70 (ZAP70) were more active in ET. Although not meeting the level for statistical relevance, it is remarkable that the activity of all Tyr kinases analyzed in this profiling was at least slightly up-regulated in ET-derived lymphocytes (normalized median kinase statistic (s) > 0.25). For example, the activity of Fms like tyrosine kinase 3 (FLT-3), Proto-oncogene tyrosine-protein kinase FER (Fer) and Janus kinase 1 (Jak1b) was markedly increased in lymphocytes of ET compared to UT (s > 0.4). However, in this regard, the Q_sp_-value was clearly below the statistical relevance threshold. All data of the Tyr kinase statistic can be found in detail in Table S3.

**Table 1:** Kinase statistic of the top 10 ranked Tyrosine kinases based on Median final score (Q).

Kinase Name	Median Final score (Q)	Specificity Score (Q_sp_)	Median Kinase Statistic (s)
FGFR3	3,80	2,40	0,58
FGFR2	3,62	2,42	0,57
FGFR4	3,34	2,00	0,49
FGFR1	2,63	1,41	0,47
ZAP70	2,50	1,61	0,43
FLT3	1,98	0,93	0,46
Met	1,90	0,98	0,44
CSK	1,79	0,92	0,42
FAK1	1,65	0,58	0,42
Syk	1,61	0,71	0,41

The constituents of the kinase statistic: Median Final Score (Q), Specificity Score (Q_sp_) and Normalized kinase statistc (s) are displayed as columns and the top ranked kinases are presented in the rows. The final ranking of the kinases was based on Median Final Score (Q).

### Decrease of Ser/Thr kinase activity in ET-derived lymphocytes

Based on the detailed pattern of differential peptide phosphorylation for all individual samples (Fig. 1d), the mathematical mean of peptide phosphorylation including all STK substrates for both experimental groups is depicted in Fig. 2c. The specific profile for the distinct substrates used to predict the reduced activity of the top-five ranked kinases in ET-derived lymphocytes is reflected by the heat maps shown in Fig. 2d. Kinase profiling revealed a statistically relevant down-regulation of the activity of nine Ser/Thr kinases in the lymphocytes of athletes (ET) (Q_sp_ > 1.3) (Fig. 3b). In particular, the top ranked Ser/Thr kinases (cGMP-depentent protein kinases 2 (PKG2), inhibitor of nuclear factor kappa B kinase subunit alpha (IKKα), PKG1, calcium-/calmodulin dependent protein kinase type IV (CaMK4), protein kinase A alpha (PKAα), ribosomal protein S6 kinase beta-1 (p70s6Kβ), protein kinase X-linked (PRKX), protein kinase C alpha (PKCα) and PKCδ were demonstrated to be less active in ET. Focusing on the extent of regulation indicated by normalized kinase statistic (s), the inhibitor of nuclear factor kappa a kinase subunit (IKKα), CaMK4 and casein kinase 2 alpha (CK2α1) showed the strongest decrease of activity in ET (s < −0.5). The kinase statistic of the top 10 ranked Ser/Thr kinases is presented in Table 2. Although not statistically significant, the activity of at least 14 other Ser/Thr kinases (e.g. AMPKα1, CaMK2α and PKCε) was markedly reduced in ET (s < −0.30). Moreover, the activity of some members of prominent Ser/Thr kinase families was at least slightly increased. For example, a minor up-regulation of kinase activity was demonstrated for members of the stress-responsive p38- and ERK families in the group of athletes (ET). However, in this respect, the specificity score (Q_sp_) was below the statistical relevance threshold. All data of the Ser/Thr kinase statistic can be found in detail in Table S4.

**Table 2:** Kinase statistic of the top 10 Serine/Threonine kinases.

Kinase Name	Median Final score (Q)	Specificity Score (Q_sp_)	Median Kinase Statistic (s)
PKG2	4,08	2,61	−0,42
IKKα	4,00	2,06	−0,72
PKG1	3,92	2,65	−0,39
CaMK4	3,92	2,43	−0,52
PKAα	3,88	2,67	−0,37
p70S6Kβ	3,67	2,27	−0,43
PRKX	3,40	1,99	−0,34
PKCα	2,92	1,73	−0,37
CK2α1	2,51	0,92	−0,51
PKCδ	2,38	1,33	−0,34

The constituents of the kinase statistic: Median Final Score (Q), Specificity Score (Q_sp_) and Normalized kinase statistc (s) are displayed as columns and the top ranked kinases are presented in the rows. The final ranking of the kinases was based on Median Final Score (Q).

### Phylogenetic kinase mapping revealed that cluster a of differently regulated ser/thr kinases belongs to AGC-kinase family

**Figure 4:**
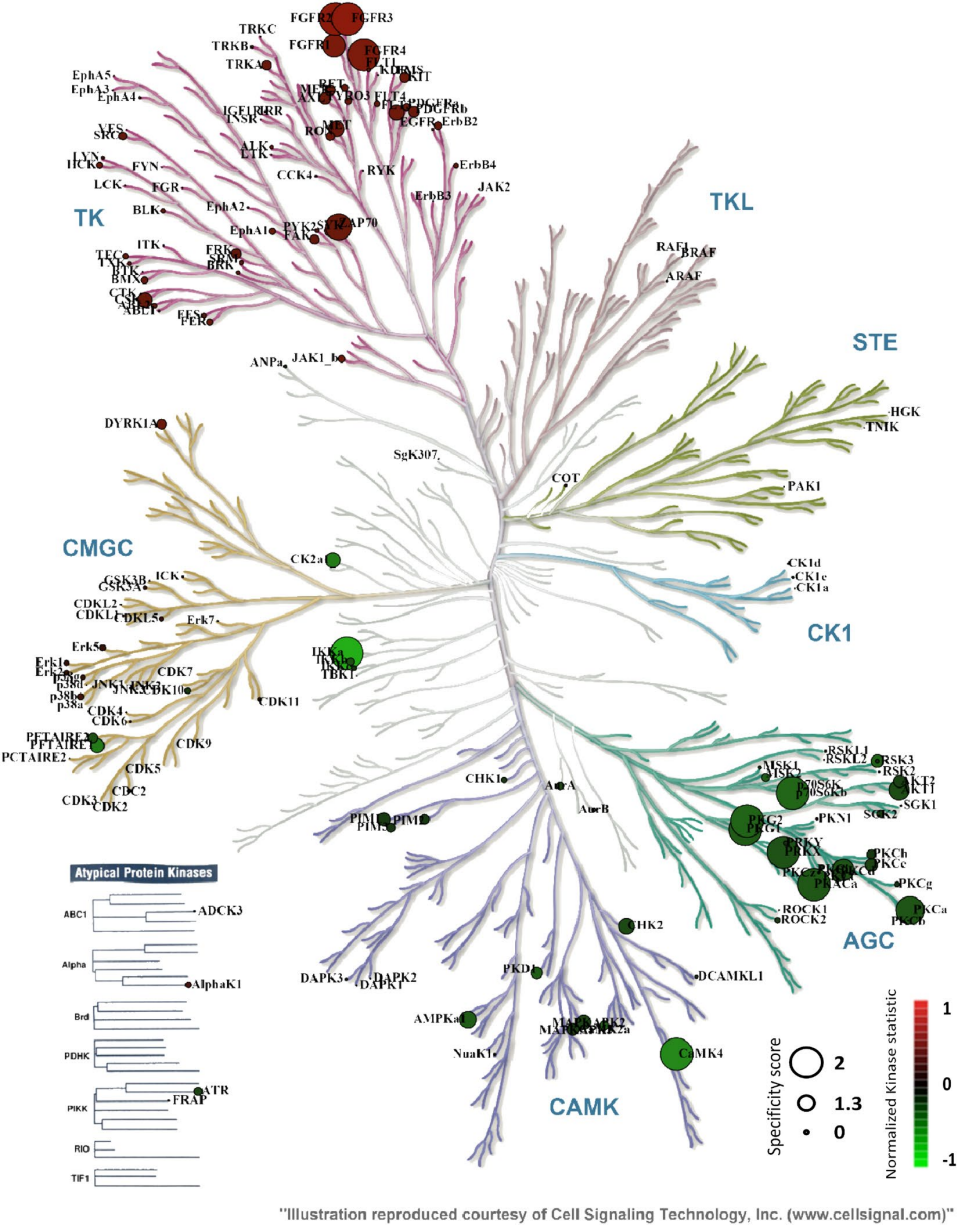
KinMap: Phylogenetic kinome tree. Phylogenetic kinase mapping illustrates the phylogenomic distribution of the kinases analyzed in tyrosine and serine/threonine activity profiling and their regulation. Tyr kinases, whose activity differed between ET and UT, are predominantly clustered in the family of Fibroblast growth factor receptors (FGFRs) and Syk-family (ZAP70). A striking feature was the clustering of many differentially regulated Ser/Thr kinases in the AGC-kinase familiy. The differences in the activity of Ser/Thr kinases (predominantly down-regulated in ET) and tyrosine kinases (all at least slightly up-regulated in ET) are clearly shown in this figure. The circle size was based on Specificity score (0 to 2, 2 being largest). The default was set to coloring according to Normalized kinase statistic (s). The coloring scale indicating the changes in kinase activity ranges from −1 (strong decrease of kinase acitivity in ET vs. UT, green color) over 0 (no change of kinase activity in ET vs. UT, black color) to +1 (strong increase of kinase acitivity in ET vs. UT, red color). AGC: protein kinase A, G and C families; ZAP70: Zeta-chain-associated protein kinase 70; ET = endurance trained athletes; UT = untrained subjects.

Phylogenetic kinase mapping showed that seven (PKG2, PKG1, PKAα, p70S6Kβ, PRKX, PKCα and PKCδ) (Q_sp_ > 1.3) most differently regulated Ser/Thr kinases in ET are part of the AGC-kinase family (Fig. 4). In contrast, only one member, each of CAMK-family (CaMK4) and IKK-family (IKKα) belongs to the group of most strongly modulated Ser/Thr kinases. CK1 family, STE- and TKL-group had no members that differed highly specific between ET and UT. Moreover, the most specifically (Q_sp_ > 1.3) regulated members of the Tyr kinase family were part of the fibroblast growth factor receptor (FGFR3, FGFR2, FGFR4, FGFR1) and SyK familiy (ZAP70).

### Network analysis reveals functional associations between differentially regulated Tyr and Ser/Thr kinases in ET-derived lymphocytes

**Figure 5:**
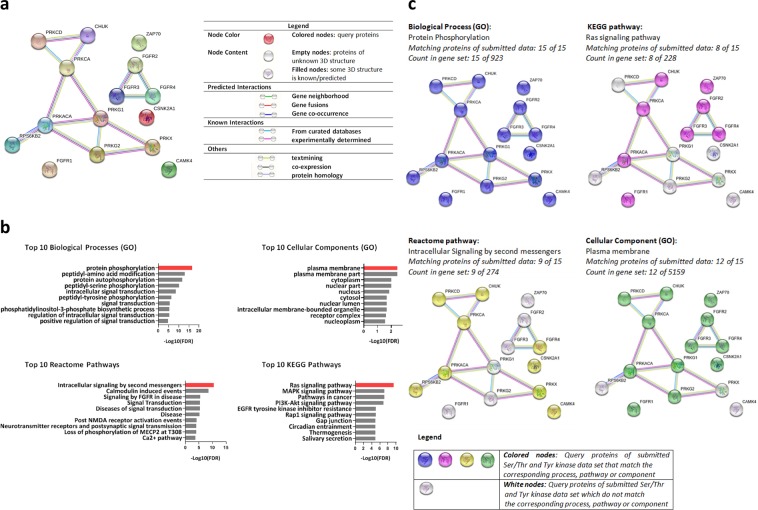
Network interactions and Functional enrichment analysis. (**a**) Network interactions. The network view generated by String Version 11.0 summarizes the network of predicted associations for tyrosine (Thr) and serine/threonine (Ser/Thr) kinases, which were associated with chronic endurance training and showed significantly increased/reduced kinase activity in lymphocytes of ET (Q_sp_ > 1.3). Kinase activity in our study was measured under conditions of physical rest. Under the conditions of an acute bout of exercise, the functional associations between the proteins may differ. The protein kinases were represented by the colored network nodes. The edges show the predicted functional associations. The colored lines (up to 8) that built an edge represent the existence of eight different types of evidence in predicting the associations. A more detailed characterization of the associations between the kinases is given in the graphic legend. (**b**) Functional enrichment analysis. The network analysis was performed for Tyr and Ser/Thr kinases, whose activity differed (Q_sp_ > 1.3) between endurance trained athletes (ET) and untrained participants (UT) in kinase profiling. The UniProt IDs of the candidates were submitted to STRING Version 11. for identification of the 10 most highly overrepresented (A) Biological processes (GO), (B) Cellular Components (GO), (C) Reactome pathways and (D) KEGG pathways. The values for false discovery rate (FDR) were log transformed and indicated by the length of the bars. The highest ranked term is marked by red color. For a more detailed description of its characteristics, see Fig. 5c. For example, IKKα (CHUK), FGFR1-4, PKAα (PRKACA), PKCα (PRKCA) and ZAP70 match the highest-ranked term Ras signaling pathway (KEGG pathway). A full overview of the kinases that match the corresponding terms is shown in the supplementary information (Table 6–9). (**c**) Network view of the kinases that match the highest ranked Biological process (GO), KEGG pathway, Reactome pathway and Cellular component (GO). Query Tyr and Ser/Thr kinases of the submitted data set that match the highest rated Biological process (GO), KEGG pathway, Reactome pathway and cellular component (GO) are shown as colored nodes. Kinases not being part of the corresponding pathway or component were marked by white nodes.

The UniProt IDs of the Tyr and Ser/Thr kinases whose activity differed (Q_sp_ > 1.3) between ET and UT were submitted to STRING database to identify network interactions. Overall, functional enrichment analysis revealed that the submitted kinases were at least partially biologically connected (Protein-protein-interaction (PPI) enrichment p = 0.0001). A detailed network view which summarizes the network of predicted functional associations between the kinases is given in Fig. 5a. The predicted functional associations between FGFR2, FGFR3 and FGFR4 were characterized by protein homology, co-expression, text mining and database evidence. Furthermore, IKKα (CHUCK) and the seven Ser/Thr kinases of AGC-family were biologically connected e.g. as shown by experimentally determined interactions. For ZAP70, FGFR1, CAMK4 and CK2α1 (CSNK2A1), no functional associations with the other kinases were predicted. The ten most relevant Biological processes (GO), KEGG- and Reactome pathways and Cellular components (GO) sorted by false discovery rate (FDR) were presented in Fig. 5b. A full overview of the kinases that match the corresponding term is shown in the supplementary information (Tables 6–9). Further, a detailed network map of the Tyr and Ser/Thr kinases, which match the highest-ranked term, is presented in Fig. 5c. Among the top 10 biological processes (GO) that were over-represented in the submitted data are protein phosphorylation, peptidyl-amino-acid modification and protein autophosphorylation. As shown in Fig. 5c, all query kinases were involved in the protein phosphorylation process. The plasma membrane, plasma membrane part and the cytoplasm were identified to be the most highly enriched cellular components (GO) in the submitted kinase set. Noticeably, all of the query Tyr and Ser/Thr kinases except CAMK4, PRKX and p70S6Kβ (RPS6KB2) were part of the plasma membrane. The highest scored KEGG pathways are linked to Ras signaling pathway (e.g. MAPK signaling pathway, PI3K-Akt signaling pathway) as well as pathways in cancer. Specifically, each of the five Tyr kinases (FGFR3, FGFR2, FGRFR4, FGFR1 and ZAP70) and three Ser/Thr kinases (IKKα: CHUCK, PKCα: PRKCA, PRKACA: PKAα) which were differently active in ET, matched the Ras signaling pathway. The Reactome pathways include pathways related to intracellular signaling by second messengers and calcium signaling. In particular, six Ser/Thr kinases of the AGC-family, CAMK4, CK2α1 which activity was decreased in ET and one up-regulated Tyr kinase (FGFR4) fit the Intracellular signaling by second messenger’s pathway.

### Protein expression of selected Tyr and Ser/Thr kinases

**Figure 6:**
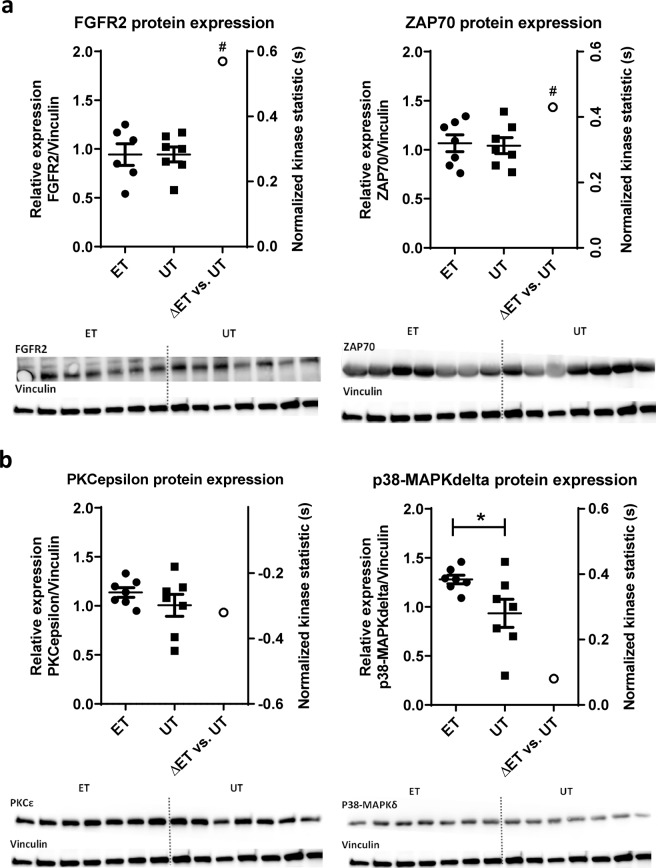
Relative protein concentrations and normalized kinase statistic (s) of selected candidates of the Tyr (FGFR2, ZAP70) and Serine/Threonine (PKCepsilon, p38δ) kinase activity profiling. All blots were performed on a single membrane. After protein transfer (Fig. S1A), the membrane was cut at 55 kDa to obtain an upper section (>55 kDa) and a lower section (<55 kDa) (Fig. S1B). Both parts were then incubated with primary antibodies for the respective protein of choice according to their molecular weight in a sequential arrangement. A stripping step was performed after each membrane development step. Corresponding full-length blots and additional information are included in the supplementary information file (Figs. S1–S6). The upper section of the membrane was incubated in the following order: 1. PKCε (82 kDa) (Fig. S2); 2. ZAP70 (70 kDa) (Fig. S3); 3. FGFR2 (110 kDa) (Fig. S4); 4. Vinculin (130 kDa) (Fig. S5). The lower section was incubated with p38δ (Fig. S6). (**a**) Protein expression (left y-axis) and normalized kinase statistic (s) (right y-axis) of selected tyrosine kinases. The bars, shown in the top (left y-axis), represent the data from the densitometry analysis (mean ± SD) (n = 7/group). The protein levels of FGFR2 and ZAP70 were normalized to the corresponding Vinculin control and expressed relative to the protein level of the untrained group. Representative western blot analysis revealed no significant differences between the experimental groups (ET vs. UT). The blots of FGFR2 and ZAP70 are each grouped with vinculin which was measured in a different region of the same membrane using a different exposure condition in terms of antibody dilution, time and substrate for signal development (for details see supplementary information). Additionally, the results of normalized kinase statistic (s) were added on the right-axis for FGFR2 and ZAP70. The activity of both kinases was increased in lymphocytes of endurance-trained athletes (Q_sp_ > 1.3). (**b**) Protein expression (left y-axis) and normalized kinase statistic (s) (right y-axis) of selected serine/threonine kinases. The bars, shown in the top (left y- axis), represent the data from the densitometry analysis (mean ± SD) (n = 7/group). The protein levels of PKCε and p38δ were normalized to the corresponding Vinculin control and expressed relative to the protein level of the untrained group. The relative protein level of p38δ was increased in ET (p ≤ 0.05). No significant differences between the experimental groups were demonstrated for PKCε (ET vs. UT). The blots of PKCε and p38δ are each grouped with vinculin which was measured in a different region of the same membrane using a different exposure condition in terms of antibody dilution, time and substrate for signal development (for details see supplementary information). In addition, the results of normalized kinase statistic (s) were added on the right-axis for PKCε and p38delta. The activity of PKCε was decreased in ET-derived lymphocytes (Qsp > 1.3). The activity of p38δ was slightly but not significantly increased in lymphocytes of endurance-trained athletes. PKCε: PKCepsilon; p38δ: p38delta; ET: endurance trained athletes, UT: untrained subjects.

In order to analyze if the differences of Tyr and Ser/Thr kinase activity in ET could be related to protein expression at basal level, representative Western blot analyses for selected candidates of the profiling were performed. Here, no significant differences between the experimental groups were found for the relative protein levels of FGFR2, ZAP70 and PKCε (Fig. 6). With respect to stress-responsive p38 delta mitogen-activated protein kinase (p38δ) the relative protein level was increased in ET (p < 0.05). Raw data for the relative protein density of each protein analyzed by Image J and normalization to Vinculin and UT are presented in detail in Table S5.

## Discussion

The present study constitutes an advance for the understanding of the effects of endurance exercise training on lymphocyte physiology at the kinase activity level. Actually, this is the first study demonstrating that the activity of multiple Tyr and Ser/Thr kinases in human lymphocytes was affected by endurance training status. Notably, the activity of a number of prominent tyrosine kinases such as members of FGFR family and ZAP70 was increased in endurance trained athletes (ET), whereas the activity of several Ser/Thr kinases e.g. PKAα, IKKα and CaMK4 was decreased. Phylogenetic mapping and network analysis revealed close functional associations between several differentially regulated kinases in ET-derived lymphocytes. Especially tyrosine receptor kinases of the FGFR-family and serine/threonine kinases of the AGC-family represent key components within the kinase network of human lymphocytes mediating the long-term effects of endurance exercise training. Mainly, KEGG pathways which are related to Ras signaling pathway as well as reactome pathways linked to intracellular signaling by second messengers and calcium signaling were found to be enriched in the present data.

Actually, this is the first study indicating an effect of chronic endurance exercise training on the kinase activity of lymphocytic FGFRs and ZAP70. Since we could show no differences in protein expression levels between the two experimental groups with respect to both kinases, other mechanisms seem to be responsible for the increased activity of these factors in lymphocytes of ET. FGFRs are receptor tyrosine kinases that upon binding to their respective ligands FGFs stimulate cellular proliferation, migration, and differentiation^27–29^. They are known to enable T-cells to respond to FGFs expressed as reaction to inflammation or injury and may thereby be primed for context-dependent reactions^28,30,31^. There is evidence that acute prolonged intense bouts of endurance exercise, such as those often experienced by elite marathoners and triathletes, can trigger pro-inflammatory immune responses by secretion of inflammatory cytokines as well as growth factors stimulating the migration of lymphocytes into the tissue^32–36^. Accordingly, it is speculated that the augmented activity of FGFRs on the plasma membrane of ET-derived lymphocytes could at least partly be related to the exercise-induced migration of immune cells to the sides of injury and inflammation promoting tissue repair and cellular adaption’s to exercise training. Besides, the up-regulation of the receptors of the FGF-family might be a consequence of altered levels of receptor ligands. There is some evidence that regular training is associated with lower serum concentrations of fibroblast growth factor which in turn would induce an increase of receptor sensitivity^37^. Furthermore, an increased basal activity of the tyrosine kinase ZAP70 in ET-derived lymphocytes was found. Interestingly, ZAP70, which is part of the T cell receptor and predominantly expressed near the surface membrane of T and NK cells, plays an essential role in the modulation of adaptive immune response^38–40^. After TCR activation by antigen presenting cells, ZAP70 contributes to the regulation of cellular motility, adhesion, proliferation, differentiation and lymphokine production^41–43^. ZAP70 is also involved in the activation of primary B cells^38,44^. Hence, it is assumed that the increased activity of ZAP70 in the ET-derived lymphocytes might reflect enhanced immune competence and ongoing cellular adaptation mechanisms that prepare the athlete’s immune system to combat potential stressful stimuli during exercise.

Divergently to the tyrosine kinases, Ser/Thr kinase activity was attenuated for all kinases identified as statistically relevant in ET. Particularly, IKKα and CaMK4 showed the strongest decrease in kinase activity as assessed by normalized kinase statistic in ET versus UT. Functionally, IKK_α_ represents a catalytic subunit of the IKK complex contributing to the activation of the nuclear factor kappa-light-chain-enhancer of activated B cells (NF-κB) via the canonical and non-canonical pathway^45,46^. Since NF-κB controls several inflammatory genes and is found to be persistently active in inflammatory diseases^47^, endurance exercise training might mediate anti-inflammatory effects by the down regulation of IKK_α_ activity. Moreover, it was demonstrated that isoforms of CaMK are implicated in mediating exercise signaling of skeletal muscle^9,11,48^. Upon activation by the binding of Ca^2+^/Calmodulin (CaM), CaMK4 is translocated into the nucleus, where it modulates the activity of numerous transcription-related factors, such as cyclic-AMP response- element-binding protein (CREB), histone deacetylase 4 and monocyte enhancer factor 2 A (MEF2)^49–52^. These key factors play pivotal roles in inflammation, the development and function of the immune response by regulating cytokines secretion, cell signaling and T cell differentiation^52^. In chronic autoimmune diseases such as systemic lupus erythematodus (SLE), surplus activation of CAMK is associated with disruptions of the balance of TREGs and TH17cells and suppression of IL-2 production^52–54^. Since aberrant activation of CAMK4 contributes to T lymphocytes abnormalities^54^, the reduction of kinase activity by chronic endurance training may improve the functionality of the adaptive immune response.

Likewise, the group of AGC-kinases including the cAMP-dependent protein kinase (PKA), the cGMP-dependent protein kinase (PKG) and the protein kinase C (PKC) family was affected by chronic endurance training. In support with these findings, the regulation of PKA, PKC_α_ and PKC_δ_ has already been described in human and murine skeletal muscle after acute endurance and resistance exercise^9,11^. Further, cardiac mitochondrial proteome seems to adapt highly dynamic to exercise training due to the action of some AGC kinases such as PKA and PKG^8,10^. It is suggested that the simultaneous change in the activity of multiple kinases of the AGC-kinase family in lymphocytes represents an adaptation to chronic endurance training. The modulation of the these kinases affects several cellular functions^55^. PKA represents a key modulator of immune response by inhibiting the early as well as late phase of antigen-induced T and B cell activation^56–58^. The induction of the cAMP signaling pathway leads to PKA activation in lymphoid cells which is known to abolish T and B cell proliferation and decreases the cytotoxicity of natural killer (NK) cells^59^. Since the regulation of PKA activity is anticipated as purposeful target in several autoimmune disorders^56^, endurance exercise training might provide a beneficial effect for human’s health by affecting T- and B-Cell activation and proliferation via a reduction of basal PKA_α_ activity. Similarly, the activity of PRKX, a novel cAMP-dependent Ser/Thr protein kinase member, was also down-regulated in ET-derived lymphocytes. While the role of PRKX in lymphocytes is almost unknown, it has been shown to play a role in angiogenesis and myeloid cell differentiation^60–62^. Moreover, PRKX binds to enzymes such as Pin-1, Magi-1 and Bag-3, which regulate cell differentiation, proliferation, apoptosis and tumor genesis^61^. Therefore, PRKX may play a central role in the regulation of lymphocyte function in endurance athletes. Furthermore, the activity of p70S6K_β_, which belongs to the S6Kfamily of AGC kinases, was reduced in lymphocytes from ET. Kinases of the S6K family represent key substrates of mTOR and affect several important celluar functions such as cell growth, proliferation and differentiation by modulating the ribosomal biogenesis, protein synthesis and metabolism^63,64^. Besides, p70S6K represents a major regulator of glucose metabolism in T cells and mediates TNF-alpha induced insulin resistance via phosphorylation of IRS1 on several serine residues^65–67^. Since over-activation of S6K family members is associated with metabolic disorders, the reduced activity might provide beneficial effects for the athlete’s health^68^.

As part of the adaptive immune response, PKCs function as key cytoplasmic signal transducers in the regulation of signaling pathways finally leading to the expression of key genes^69^. The activity of PKC_α_ was diminished in lymphocytes of ET. Studies indicate that PKC_α_ is involved in mediating the T cell receptor (TCR)-induced activation of IKK and NF-κB, which represents an important key transcription factor of the immune response and inflammation^70^. Beyond, PKC_α_ modulates TCR down regulation^71^. Accordingly, reduced activity of PKC_α_ in the lymphocytes of athletes under conditions of physiological rest might promote an anti-inflammatory immune response via abolishing NF-kB signaling and favoring activation of T-lymphocytes by up-regulating TCRs. Moreover, recent studies revealed also a regulatory role of some PKC isoforms in the nucleus as chromatin-associated kinases^69^. Especially PKC_δ,_ is known to function as an epigenetic enzyme in B- and T-cells^69,72,73^. Therefore, it appears possible that a lower basal activity of PKC_δ_ in athletes might cause changes even at the epigenetic level.

The activities of PKG2 and of PKG1 were decreased in lymphocytes of the ET group. So far the functional role of PKGs in lymphocytes has been poorly studied. Since several protein targets for PKG phosphorylation are involved in the regulation of cellular calcium, it can be assumed that the down- regulation of kinase activity influences the calcium metabolism in ET-derived lymphocytes^74,75^. Further, activation induced-cell death implicates the induction of PKGs in T-lymphocytes^76^. Consistent with studies showing that lymphocyte apoptosis sensitivity is related to training status, a reduction of PKG activity may mediate anti-apoptotic effects in the athlete’s immune system^26,77^.

Causally, we can only speculate about the mechanisms that could have led to lower basal activity of AGC kinases. The measurement of the protein expression of a representative PKC variant suggests that the protein level is not responsible for the lower kinase activity in lymphocytes from ET. Therefore, future research should clarify whether reduced activity may be due to regulatory factors, such as second messengers and intracellular calcium which are known to be highly responsive to exercise^78–80^. This seems conceivable since a previous study by our group demonstrated an enhanced availability of the free intracellular calcium concentration in lymphocytes of mice in response to regular endurance training^79^. Thus, down-regulation of the kinase activity of PKC variants may represent a negative feedback mechanism due to chronic exposure to high calcium levels acting as an important co-factor of PKC activation. Moreover, autophosphorylation as well as interactions between the kinases themselves may play a role. For example, a lower activity of PKC_α_ may have resulted in down-regulation of IKK_α_ among others^70^.

Although there were no statistically significant differences in p38_δ_ kinase activity between athletes and untrained individuals, we decided on measuring protein expression because of p38 MAPKs’ high relevance as an exercise-responsive key regulator for protein synthesis and inflammatory responses^9,11^. It is suspected that the increased basal expression of p38δ in lymphocytes from ET may encourage mTOR activation by acute exercise stress which represents a pivotal factor in bridging immune and metabolic signals that control the differentiation, proliferation and survival of lymphocytes^81^.

Finally, we analyzed the interactions of training-sensitive Tyr and Ser/Thr kinases by showing their cellular distribution and involvement in signaling pathways. The enrichment of some kinases in the biological processes and reactome pathways has already been discussed above. Cellular component (GO) enrichment analysis suggests that especially kinases that are part of the plasma membrane, such as certain receptor tyrosine kinases as FGFRs or ZAP70, are predominately sensitive to modulations in kinase activity by chronic endurance training. Interestingly, KEGG-signaling pathways such as Ras-signaling and MAPK-signaling pathway were over-represented among the statistically relevant regulated kinases. Particularly, tyrosine receptor-linked kinases marked by increased activity in ET-derived lymphocytes such as FGFRs are responsible for activation of Ras-MAPK, whereas Ser/Thr kinases such as IKK_α_ or PKC_α_, whose activity was decreased, represent down-stream targets of the Ras signaling pathway^82,83^.

So far, we did not present any experiments revealing the mechanistic details or the functional consequences of the up- or down-regulation of the activity of those distinct kinases. Thus, our results for network interactions and functional enrichment derived by String are limited by the fact that most associations cannot be specified precisely in terms of their mode interaction or the cellular conditions under which they occur^84,85^. However, we would like to take the possibility to link our herein reported findings with data from a previous study by our group to discuss how phosphorylation could affect downstream signaling pathways^26^. In our previous study, we analyzed the expression of micoRNAs (miRs) and apoptotic genes in the lymphocytes of the same subjects^26^. At baseline, lymphocytes of ET were characterized by an up-regulation of the anti-apoptotic target gene XIAP), which was associated by a down-regulation of one of its regulatory pro-apoptotic miR-23a. Moreover, a trend could be observed for an increase of BCL2 expression of ET^26^. Among other factors such as miRs, the expression of the anti-apoptotic genes namely XIAP and BCL2 is regulated via the transcription factor NF-kappa-B^86,87^. Interestingly, Ras signaling pathway including the kinases IKKα, FGFR 1–4, PKAα, PKCα and ZAP70 (Fig. 5c) is linked to the activation of the NF-kappa-B signaling pathway which stimulates the expression of anti-apoptotic proteins such as BCL2 and XIAP. Therefore, the increased expression of those -amongst others - anti-apoptotic genes may be related to differential kinase activity in lymphocytes of endurance-trained individuals. This all points to phenotypic changes of lymphocytes due to endurance training. However, future research is needed to experimentally validate the functional relationships between the proteins and to specify the role of each single kinase.

Importantly, the majority of the Tyr and Ser/Thr kinases, which were affected by aerobic endurance training status in the present study such as ZAP70, FGFRs, CaMK4, IKK_α_ and some members of AGC kinase family are also used as pharmacological targets for kinase inhibitors with respect to the treatment of immunological and autoimmune diseases, cancer and viral infection^88–91^. For example, the regulation of cAMP/PKA pathway is essential to protect against immunological overshoot by controlling TCR signaling^92^. Under disease conditions, such as chronic infections or cancer, T cells have high cAMP levels leading to surplus down regulation of TCR signaling^58^. COX (cyclooxygenases) inhibitors are known to negatively regulate the cAMP/PKA signaling pathway by inhibiting the action of COX, which results in a decrease of intracellular cAMP and down regulation of PKA activity. Despite their efficiency, the use of COX inhibitors can cause unwanted side effects. Therefore reducing their dose and developing novel drugs targeting the pathway at a different stage may be beneficial^58^. Our results indicate that endurance training reduces the activity of PKAα. Moreover, endurance training increases the activity of ZAP70 which may also counteract TCR down regulation. Therefore, the synergistic use of endurance training supplementary to pharmacological kinase inhibitors or the development of a kinase cocktail that mimics the positive effects of endurance training in human immune cells would be conceivable^9^.

Since this study has a pilot character, and this is the first study ever that deals with adaptations of kinase activity in immune cells to exercise training using a kinase profiling approach, we have decided to analyze lymphocytes as total population. We suggested that analyzing a mixed lymphocyte population represents best how the kinase activity differs at diverse physiological conditions. In the athlete´s body, the single lymphocyte subpopulations are not isolated and there is an intense cellular crosstalk which affects kinase activity in the individual cell-types including the lymphocyte subpopulations. Given that, the same amount of lymphocyte protein content was used for each subject. Therefore an effect of different lymphocyte cell numbers on the kinase activity profiling can be excluded. However, a limitation of the present study is the lack of evaluation of kinase activity in lymphocyte subpopulations. Thus, we cannot state to what extent the phosphorylation pattern differs between lymphocyte subpopulations. Our previously published results revealed no significant differences for CD19^+^ and CD4^+^ lymphocytes as well as for the CD4/CD8 ratio between endurance-trained (ET) athletes and un-trained (UT) participants^26^. However, only the absolute numbers of CD3^+^ and CD8^+^ lymphocytes were significantly decreased in the athletes group^26^. The biological function (Table S10) and expression of the analyzed kinases may differ in lymphocyte subpopulations. Basically, the level of kinase activity is not directly related to its expression. However, a possible effect of the results of some kinases such as CAMK4, which are predominantly present in T cells, cannot be ruled out. Given that the activity of kinases in subpopulations may vary, this could potentially affect the results. Furthermore, the study is limited by the fact that the kinase profiling was based on a prediction method processing the measured peptide phosphorylation patterns by using *in vitro/in vivo* and literature databases. Nevertheless, the differences in the measured protein phosphorylation patterns of endurance trained athletes and untrained individuals were clearly visible (Tables S1 and S2) and as *in vitro/in vivo* databases were assigned the highest priority, the validity of the method can be considered good. Finally, we cannot clearly state to what extent the differences in body composition as indicated by lower total body fat and body mass index (BMI) as well as nutritional factors may contribute to alterations in Tyr and Ser/Thr kinase activity in ET.

In conclusion, endurance exercise training seems to have a major impact on the regulation of the basal activity of multiple Tyr and Ser/Thr kinases in human lymphocytes and thus, may provide beneficial effects for health by improving the adaptive immune competence. The use of a highly sensitive peptide-based kinase activity profiling approach has elucidated the complexity of adaptations to endurance exercise training in human lymphocytes, which seems also to be of great importance for the regulation of stress-sensitive immunological signaling pathways. Next, future studies should clearly identify the mechanisms that may have been responsible for adapting lymphocytic kinase activity to endurance exercise training. Furthermore, the clinical benefits of modulating the Tyr and Ser/Thr kinase activity profile of lymphocytes by exercise should be elucidated. In this regard, the kinase activity and health effects of a moderately-trained exercise group should also be investigated. Hereby, we believe that the study of exercise-induced adaptations of kinase activity in immune cells and its mechanisms could represent an advance in developing novel strategies for the generation of effective kinase inhibitors for immunological applications.

## Materials and Methods

The current work is based on a recent previous study of our group^26^. The lymphocyte samples were obtained from the same subjects. We refer to this publication for data regarding anthropometric and physiological characteristics, leukocyte cell count and lymphocyte subpopulation.

### Ethical approval

This study was carried out in accordance with the Declaration of Helsinki. The study procedure was approved by the local ethics committee of the Justus-Liebig-University Giessen (Germany). Informed consent of all participants was obtained before study participation.

### Study design

**Figure 7:**
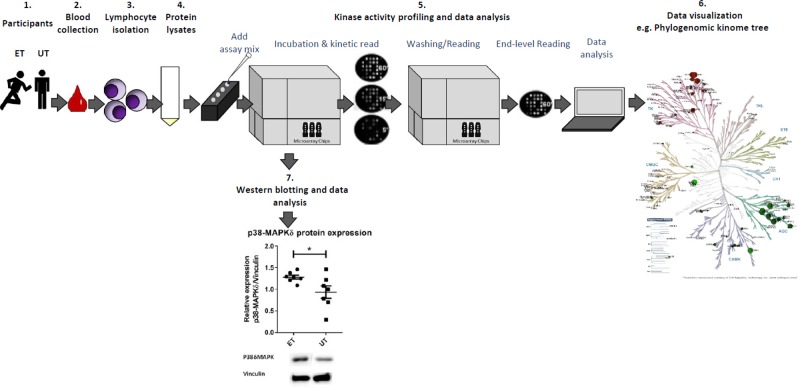
Experimental design of the kinase activity profiling of human lymphocytes in endurance trained athletes (ET) and untrained individuals (UT). 1. The subjects were divided into two experimental groups. One group (ET) consisted of highly trained marathon runners and triathletes (sex: male, age: ≥18 ≤ 45 years, VO_2max_ ≥ 59 ml/kg*min^−1^), while the other group (UT) included non-athletes (VO_2max_ ≤ 45 ml/kg*min^−1^) of the same sex and age. 2. Venous blood collection was carried out under conditions of physical rest. 3. Lymphocytes were isolated from whole blood by immunomagnetic negative selection method. 4. Cell lysis and protein quantification were performed. 5. The assay mix containing the lymphocyte protein was applied to the tyrosine (Tyr) and serine/threonine (Ser/Thr) PamChip® microarray system. Kinase activity profiling and data analysis were performed using Evolve software and Bionavigator Analysis tool (both Pamgene). 6. Data visualization was conducted by using PamApp, KinMap*beta* and STRING Version 11. 7. Protein expression of selected kinase candidates of the Tyr and Ser/Thr activity profiling was analyzed by Western blotting.

Participants were medically examined for their unrestricted participation in sports and the anthropometric data were collected. An endurance exercise capacity testing was performed on the treadmill using a continuous incremental exercise protocol as recently explained in detail^26^. Subsequently, subjects were classified as either endurance trained (ET) or untrained (UT) based on their maximum relative oxygen uptake (VO_2max_) and further inclusion criteria. At the time interval of at least 7 days after the exercise testing, standardized venous blood collection was performed under resting conditions. Protein lysates were obtained from lymphocytes isolated from whole blood. A methodological overview of the experimental design of the kinase activity profiling is depicted in Fig. 7.

### Participants and testing of endurance exercise capacity

Aerobic trained marathon runners and triathletes (ET, n = 12) and untrained volunteers (UT, n = 13) were acquired. The maximum oxygen uptake and maximum running speed were measured using an incremental exercise protocol on the treadmill (h/p/cosmos quasar 4.0; h/p/cosmos Sports & Medical GmbH, Nussdorf/Traunstein, Germany). The initial running speed was adjusted at 6 km/h increasing every 2 min by 2 km/h until the participants‘ volitional exhaustion. The respiratory parameters were obtained by the Innocor System (Innovision, Glamsbjerg, Denmark). Participants were eligible for the aerobic trained group if they reached a VO_2max_ ≥ 59 mL/kg*min^−1^ in the mandatory endurance exercise capacity testing. Subjects were classified as UT if they achieved a VO_2max_ ≤ 45 mL/kg*min^−1^. A detailed presentation of all inclusion and exclusion criteria and the anthropometric and physiologic characteristics is given in^26^.

### Blood collection and lymphocyte isolation

The participants were instructed to renounce strenuous physical activity 24 hours and food intake 6 hours prior to the blood collection procedure. Blood sampling was always carried out between 8 and 11 a.m. according to a standardized procedure under conditions of physical rest. In this process, a total volume of 38.1 mL peripheral whole blood was taken from the antecubital vein using Safety-Multifly Needles (Sarstedt, Nümbrecht, Germany). Lymphocytes were separated directly from whole blood through immunomagnetic negative selection method according to the manufacturer’s instructions using EasySep^TM^ Direct Human Total Lymphocyte Isolation Kit (Stem Cell Technologies, Vancouver, British Columbia, Canada). After purification lymphocytes were washed twice with phosphate buffered saline (PBS) and then centrifuged at 640 × g and 4°Celsius for 10 min. Afterwards, the supernatant was carefully discarded and the lymphocyte pellets were immediately frozen at −80 °C.

### Leukocyte cell count and flow cytometric assessment of lymphocyte subpopulations

The analysis of leukocyte cell count and lymphocyte subpopulations was performed as previously described in detail^26^. For the assessment of leukocyte cell count a semi-automated hematology analyzer (XE2100, Sysmex, Norderstedt, Germany) was used. Lymphocyte subpopulations (T-, B-cells, CD4^+^ and CD8^+^ lymphocytes) and apoptosis were measured by flow cytometry (Beckman Coulter, EPICS XL, Brea, California, USA) using antibodies against CD3 (PE), CD19 (FITC), CD4 (PE), CD8 (FITC) (Immunotools, Friesoyte, Germany). The full data of leukocyte cell count and lymphocyte subpopulation measurement is shown in^26^.

### Cell lysis and protein quantification

100 µL M-PER lysis buffer (Mammalian Extraction Buffer, #78503, #78420, #87785, ThermoFisher Scientific^TM^, Darmstadt, Germany) was added per 1 × 10^6^ cells and syringe-and-needle homogenization was performed. The lysates were incubated for 1 hour on ice, centrifuged for 15 min at 16.000 × g at 4 °C. Next, the supernatant (containing the proteins) was transferred to pre-cooled clean tubes. Aliquots of 10 µL sample volume were prepared and snap-frozen by liquid nitrogen before storage at −80 °C. Protein quantification was conducted by bicinchoninic acid assay according to the manufacturer’s instructions using Pierce^TM^ BCA Protein Assay (#23225, Thermo Fisher). Protein concentrations were measured using microplate reader TECAN Infinite®200 PRO (Tecan Trading AG, Männedorf, Switzerland).

### Tyrosine kinase activity profiling using PamChip® peptide microarrays

Tyrosine kinase (Tyr) profiles were determined using the PamChip® peptide tyrosine kinase microarray system on PamStation®12 (Pamgene, BJ´s-Hertogenbosch, The Netherlands). The PTK PamChip® Array is a flow-through microarray assay including 144 distinctive peptide sequences spotted on a porous membrane. Each peptide on the chip builds a 15-amino acid sequence representing a putative endogenous phosphorylation site which functions as a tyrosine kinase substrate. The phosphorylation of the peptides is visualized by detection of the fluorescent signal which is emitted as a result of the binding of the FITC-conjugated PY20 anti-phosphotyrosine antibody. 7.5 µg of total lymphocyte protein were used to carry out PTK activity profiling using the standard protocol supplied by Pamgene. All reagents used for PTK activity profiling were supplied by Pamgene International B.V. Initially, to prepare the PTK Basic Mix, the freshly frozen lysate was added to 4 µL of 10 x protein PTK reaction buffer (PK), 0.4 µL of 100 x bovine serum albumin (BSA), 0.4 µL of 1 M dithiothreitol (DTT) solution, 4 µL of 10 x PTK additive, 4 µL of 4 mM ATP and 0.6 µL of monoclonal anti-phosphotyrosine FITC-conjugate detection antibody (clone PY20). Total volume of the PTK Basic Mix was adjusted to 40 µL by adding distilled water (H_2_0). Before loading the PTK Basic Mix on the array, a blocking step was performed applying 30 µL of 2% BSA to the middle of every array and washing with PTK solution for PamChip® preprocessing. Next, 40 µL of PTK Basic Mix were applied to each of four arrays of three PamChips®. Then, three PamChip®4 microarray assays were run for 94 cycles. An image was recorded by a CCD camera PamStation®12 at kinetic read cycles 32–93 at 10, 50 and 200 ms and at endlevel read cycle at 10, 20, 50, 100 and 200 ms. Instrument operations as initial sample/array processing and image capture were conducted with Evolve (Pamgene) software. In a final step, data normalization and visualization were performed using BioNavigator Analysis software tool (Pamgene). Outliers due to insufficient antibody binding (UT 5) or detection error (ET7) were excluded to ensure valid analysis.

### Serine-/Threonine kinase activity profiling using PamChip® peptide microarrays

Serine-/Threonine kinase (Ser/Thr) profiles were measured using the PamChip® peptide STK flow-through microarray system on PamStation®12 (Pamgene, BJ´s-Hertogenbosch, The Netherlands). The STK PamChip® Array contains four positive control peptides and 140 serine/threonine peptides. The peptides immobilized on the PamChip® consist of a 15-amino acid sequence and correspond to a putative phosphorylation site which serves as a serine-/threonine kinase substrate. Phosphorylation activity of Ser/Thr kinases was visualized using a two-step assay method including a mixture of primary antibodies and a fluorescently (FITC-) labeled secondary antibody. 1 µg of total lymphocyte protein was used for Ser/Thr kinase activity profiling according to the standard protocol provided by Pamgene. All reagents used for the preparation of the Ser/Tyr Basic Mix and the Detection Mix were supplied by Pamgene International B.V. The STK Basic Mix was composed of the protein lysate, 4 µL 10 x PK, 0.4 µL of 100 x BSA, 4.0 µL of 4 mM ATP, 0.46 µL STK antibody mix. Total volume of the STK Basic Mix was adjusted to 40 µL by adding 27 µL distilled water (H_2_0). The detection mix (total volume 30 µL) consisted of 3 µL of 10 x antibody buffer (AB), 0.4 µL STK antibody FITC-labeled and 26.6 µL distilled water. After performing PamChip® preprocessing from cycle 1 to 30 (for details see above), the STK Basic Mix was applied to each of the four arrays of three PamChips®. Afterwards, the Pamchip® microarrays were incubated and washed for 60 cycles (cycle 30 to 90). In a second step, the Detection Mix was transferred to the PamChips®. Incubation with FITC-labeled STK antibody and image recording (after 10, 50 and 200 ms) were performed for the next subsequent 30 cycles (starting with cycle 92) and end-level read was carried out after the final cycle 124 (10, 20, 50, 100 and 200 ms). Instrument operations, data normalization and visualization were conducted as described previously (for details see above.). One outlier (UT12) due to insufficient antibody binding was excluded to ensure valid analysis.

### Upstream kinase analysis

Upstream kinase analysis was based on Tyr and Ser/Thr kinase phosphorylation patterns and automatically calculated by default using the PamApp on BioNavigator Analysis software tool. Peptides spotted on the chip were either selected by their ability to be phosphorylated by multiple kinases or by their recognition only by unique kinases. The collection of peptides provides phosphorylable substrate for the whole kinome (e.g. 518 human kinases) and allows - in principle - a full coverage for all kinases with annotated downstream substrate proteins known so far. Prediction for the differentially activated upstream kinases is carried out by a comparison of the individual determined specific pattern of substrate peptide phosphorylation for each experimental group (UT versus ET) with databases containing empirical *in vitro/in vivo* (iviv) as well as literature-based protein modifications such as HPRD, PhosphoELM, PhosphositePLUS, Reactome, UNIPROT and *in silicio* predictions (PhosphoNet, http://www.phosphonet.ca/) database. Kinases predicted by the iviv databases were given higher weightage (rank = 0). Rankings according to PhosphoNET (from 1 to 12) were sorted by their proprietary algorithms or by modeling based on kinase active sites and predictive values. Normalized median kinase statistic (s) depicts the overall change of the peptide set that represents a substrate group for the given protein kinase. The specificity score (Q_sp_) indicates the specificity of the normalized kinase statistics with respect to the amount of peptides used for predicting the corresponding kinase. The higher the Q_sp_, the less likely it is that the normalized kinase statistic could have been generated using a random set of peptides from the present data set. Q_sp_ logarithmic values > 1.3 were considered as statistically relevant. The final ranking of the kinases was based on median final score (Q), which was calculated by addition of significance score and specificity score (Q = Q_sg_ + Q_sp_).

### Phylogenetic tree and analysis of protein kinase network

To visualize the compound Tyr and Ser/Thr profiling data and functional relationships, a phylogenetic tree was created using web-based kinome tool KinMAP_beta_ (http://kinhub.org/kinmap/). Furthermore, network analysis was performed for Tyr and Ser/Thr kinases, which were differentially regulated in ET (Q_sp_ > 1.3). A network view depicting functional associations and predicted interactions was obtained by STRING version 11.0 (https://string-db.org)^85^. Additionally, STRING was used to analyze functional enrichments in kinase network.

### Western blot analysis

Western blot analysis (WB) of lysates from human blood-derived lymphocytes was carried out for a representative randomized selected number of subjects (ET: n = 7; UT: n = 7). 23 µg of each protein sample was separated using 8% sodium dodecyl sulfate polyacrylamide gradient gel (SDS-PAGE). Subsequently, the protein bands were blotted onto a nitrocellulose membrane. The membrane was washed three times for 10 min with Tris-buffered saline plus Tween20 (TBST). Next, the membrane was incubated with a horseradish peroxidase (HRP) conjugated secondary monoclonal anti-rabbit IgG antibody (#7074 S, 1:2500, Cell signaling) for p38δ (#8690, 1:500, Cell signaling), PKCε (#2683, 1:1000, Cell Signaling), FGFR2 (#ab10648, 1:1000, Abcam, Cambridge, UK), and ZAP70 (#3165, 1:500, Cell signaling) as primary antibody. An anti-mouse secondary IgG antibody (#7076 S, 1:2500, Cell signaling) was used for Vinculin (#ab18058, Abcam). Image capture was performed by western blot imaging system Intas ChemoCam Imager and analyzed by the software Chemostar (INTAS Science and Imaging Instruments GmbH, Göttingen, Germany). The band intensity was quantified by ImageJ (University of Wisconsin, USA). Protein level was normalized to the corresponding Vinculin control. Further, one sample from the untrained group was used as control (UT) for normalization. Relative protein levels were calculated by dividing each sample with control (UT). Differences in relative protein level were analyzed by two-tailed T-Test. A p-value of p ≤ 0.05 was considered as statistically significant.

### Statistical analysis

Data analysis of the anthropometric and physiological data, leukocyte cell count and lymphocyte subpopulations was performed as described before by SPSS version 23 (IBM® SPSS Statistics, IBM GmbH, Munich, Germany) and GraphPad Prism 5.01 (GraphPad Software, LA Jolla, USA)^26^. These data are presented as means ± standard deviation or standard error of the mean. A p-value of p ≤ 0.05 was accepted as statistically significant. Metric variables of the mentioned parameters were visually and mathematically checked for normal distribution using Shapiro-Wilk-Test. The statistical differences between the two groups of unequal training status were analyzed by using unpaired two-tailed t-tests for all other data. The calculation of the effect sizes (Cohen’s d) was accomplished using G*Power 3.1.9.

## Supplementary information

Supplementary information.

Supplementary Information, Table S 1.

Supplementary information, Table S2.

## Data Availability

The data that support the findings of this study are available.
