# Supplementary material for: Profiling of human lymphocytes reveals a specific network of protein kinases modulated by endurance training status
Source: Sci Rep. 2020 Jan 21;10:888. doi: 10.1038/s41598-020-57676-6 (PMC6972788; doi:10.1038/s41598-020-57676-6)

Supplementary information

Profiling of human lymphocytes reveals a specific network of protein kinases modulated by endurance training status

Katharina Alack[1], Astrid Weiss[2], Karsten Krüger[1], Mona Höret[2], Ralph Schermuly[2], Torsten Frech[1], Martin Eggert[3], Frank-Christoph Mooren[4]

1 Department of Exercise Physiology and Sports Therapy, Institute of Sports Sciences, Justus-Liebig-University, Giessen, Germany

2 Member of the German Center for Lung Research (DZL), Cardio-Pulmonary Institute (CPI), Justus-Liebig-University, Giessen, Germany

3 Center for Extracorporeal Organ Support, Department of Internal Medicine, Universitätsmedizin Rostock, Rostock, Germany

4 Witten/Herdecke University, Faculty of Health/School of Medicine, Witten Germany

Supplementary information

Table Legends

Table S1. Raw data and heatmap representing the phosphorylation intensity of each peptide substrate spotted on the Tyr Pamchip®. The lymphocyte samples of the subjects are displayed as columns and the individual peptides are shown in the rows. The heatmap was created based on the log2 transformed raw data. Data was normalized via date centering. The peptides were ranked according to their correlation factor. The values of the phosphorylation intensities were translated into colors using a color scale. The scale for the phosphorylation intensity ranges from 1.2 (highest intensity, red color) to -1.2 (lowest intensity, blue color). AU = arbitrary units.

Table S2. Raw data and heatmap representing the phosphorylation intensitiy of each peptide substrate spotted on the Ser/Thr Pamchip®. The lymphocyte samples of the subjects are displayed as columns and the individual peptides are shown in the rows. The heatmap was created based on the log2 transformed raw data. Data was normalized via date centering. The peptides were ranked according to their correlation factor. The values of the phosphorylation intensities were translated into colors using a color scale. The scale for the phosphorylation intensity ranges from 1 (highest intensity, red color) to -1 (lowest intensity, blue color). AU = arbitrary units.

Table S3. Kinase statistic of all tyrosine kinases analyzed in this study. The constituents of the kinase statistic Specificity Score (Q_{sp}), Significance Score (Q_{sg}), Mean final score (Q_{mean}), Median Final Score (Q), Mean normalized kinase statistc (s_{mean}), Median normalized kinase statistic (s), Standard deviation of Kinase statistic (SD) and the mean peptide size are displayed as columns and the kinases marked by Unit-Prot IDs are presented in the rows. The final ranking of the kinases was based on Median Final Score (Q).

Table S4. Kinase statistic of all serine/threonine kinases analyzed in this study. The constituents of the kinase statistic Specificity Score (Q_{sp}), Significance Score (Q_{sg}), Mean final score (Q_{mean}), Median Final Score (Q), Mean normalized kinase statistc (s_{mean}), Median normalized kinase statistic (s), Standard deviation of Kinase statistic (SD) and the mean peptide size are displayed as columns and the top ranked kinases marked by Unit-Prot IDs are presented in the rows. The final ranking of the kinases was based on Median Final Score (Q).

Table S5. Raw data of each protein analyzed by Image J and the normalization procedure.

*Table S6. Biological processes (GO).*This table shows which of the input kinases have an enriched term. The terms (top 20) are ranked by false discory rate. The analysis was only performed for Tyr and Ser/Thr kinases, which were associated with chronic endurance training (Q_{sp} > 1.3). All input kinases (n = 15) match the highest-ranked term protein phosphorylation. Observed gene count: number of genes in the input data list with the term assigned; background gene count: total number of genes in the background proteome with the term assigne; FDR: false discovery rate

*Table S7. KEGG pathway .*This table shows which of the input kinases have an enriched term. The terms (top 20) are ranked by false discory rate. The analysis was only performed for Tyr and Ser/Thr kinases, which were associated with chronic endurance training (Q_{sp} > 1.3). IKKα (CHUK), FGFR1-4, PKAα (PRKACA), PKCα (PRKCA) and ZAP70 match the highest-ranked term Ras signaling pathway. Observed gene count: number of genes in the input data list with the term assigned; background gene count: total number of genes in the background proteome with the term assigne; FDR: false discovery rate

*Table S8. Reactome pathway.*This table shows which of the input kinases have an enriched term. The terms (top 20) are ranked by false discory rate. The analysis was only performed for Tyr and Ser/Thr

kinases, which were associated with chronic endurance training ($Q_{sp} > 1.3$). CAMK4, IKKα (CHUK), CK2α1 (CSNK2A1), FGFR4, PKAα (PRKACA), PKCα (PRKCA), PKCδ (PRKCD), PRKX and p70S6Kβ (RPS6KB2) match the highest-ranked term Intracellular signaling by second messengers. Observed gene count: number of genes in the input data list with the term assigned; background gene count: total number of genes in the background proteome with the term assigne; FDR: false discovery rate

Table S9.Cellular component. This table shows which of the input kinases have an enriched term. The terms (top 20) are ranked by false discory rate. The analysis was only performed for Tyr and Ser/Thr kinases, which were associated with chronic endurance training ($Q_{sp} > 1.3$). IKKα (CHUK), FGFR1-4, PKAα (PRKACA), PKCα (PRKCA), PKCδ (PRKCD), PKG1 (PRKG1), PKG2 (PRKG2) and ZAP70 match the highest-ranked term plasma membrane. Observed gene count: number of genes in the input data list with the term assigned; background gene count: total number of genes in the background proteome with the term assigne; FDR: false discovery rate

*Table S 10. This table provides an overview of the most important functions of the most highly ranked protein kinases modulated by endurance training status.*The biological role of some of the kinases or specific kinase isoforms (e.g. PRKX) has not yet been clarified for lymphocytes or individual subpopulations and therefore, cannot be specified in detail due to a lack of studies. The function and phosphorylation of these kinases may differ in lymphocyte subpopulations.

Table S3

Kinase Uniprot ID	Kinase Name	Mean Specificity Score	Mean Significance Score	Mean Final Score	Median Final score	Mean Kinase Statistic	Median Kinase Statistic	SD Kinase Statitistic	Mean peptide set size
P22607	FGFR3	2,40	1,33	3,73	3,80	0,57	0,58	0,01	5,17
P21802	FGFR2	2,42	1,33	3,75	3,62	0,57	0,57	0,01	5,71
P22455	FGFR4	2,00	1,18	3,17	3,34	0,51	0,49	0,06	12,22
P11362	FGFR1	1,41	1,07	2,48	2,63	0,50	0,47	0,05	7,67
P43403	ZAP70	1,61	0,97	2,58	2,50	0,43	0,43	0,00	41,00
P36888	FLT3	0,93	0,99	1,92	1,98	0,46	0,46	0,02	9,14
P08581	Met	0,98	0,88	1,86	1,90	0,44	0,44	0,01	18,33
P41240	CSK	0,92	0,94	1,86	1,79	0,42	0,42	0,01	30,44
Q05397	FAK1	0,58	0,86	1,43	1,65	0,40	0,42	0,02	27,11
P43405	Syk	0,71	0,88	1,59	1,61	0,41	0,41	0,00	47,00
P30530	Axl	0,72	0,86	1,58	1,60	0,41	0,41	0,01	36,33
P09619	PDGFR[beta]	0,66	0,95	1,61	1,58	0,43	0,43	0,01	10,89
P04626	HER2	0,50	0,88	1,38	1,46	0,41	0,41	0,02	19,22
P16591	Fer	0,41	0,82	1,23	1,46	0,41	0,45	0,05	7,00
Q12866	Mer	0,65	0,86	1,51	1,44	0,41	0,41	0,02	32,56
P42685	FRK	0,63	0,82	1,45	1,43	0,41	0,41	0,01	31,33
P51813	Etk/BMX	0,46	0,89	1,35	1,43	0,41	0,41	0,01	22,00
Q04912	Ron	0,56	0,84	1,40	1,35	0,44	0,44	0,02	6,22
P23458	JAK1~b	0,48	0,86	1,33	1,34	0,44	0,44	0,00	3,00
P10721	Kit	0,62	0,93	1,55	1,31	0,44	0,42	0,05	8,78
P16234	PDGFR[alpha]	0,45	0,86	1,31	1,30	0,43	0,43	0,00	5,00
Q06418	Tyro3/Sky	0,44	0,81	1,25	1,29	0,40	0,41	0,02	28,44
P35916	FLT4	0,38	0,85	1,22	1,28	0,40	0,41	0,02	8,11
P42680	TEC	0,38	0,88	1,26	1,23	0,40	0,40	0,02	26,22
P17948	FLT1	0,28	0,90	1,18	1,23	0,39	0,39	0,01	12,89
P04629	TRKA	0,56	0,86	1,43	1,22	0,43	0,41	0,04	7,56
P21709	EphA1	0,43	0,83	1,25	1,22	0,43	0,42	0,01	3,60
P12931	Src	0,50	0,84	1,34	1,20	0,41	0,40	0,02	26,44
P07949	Ret	0,45	0,82	1,27	1,16	0,41	0,40	0,02	11,67
Q9H3Y6	Srm	0,32	0,78	1,10	1,16	0,39	0,40	0,01	29,22
P42681	TXK	0,26	0,77	1,03	1,14	0,37	0,38	0,04	21,78
Q9UM73	ALK	0,26	0,84	1,10	1,10	0,39	0,39	0,00	28,11
P42684	Arg	0,32	0,87	1,20	1,10	0,40	0,39	0,01	34,44
P07332	Fes	0,29	0,79	1,08	1,09	0,39	0,40	0,02	9,33
P08631	HCK	0,41	0,82	1,23	1,09	0,41	0,39	0,03	18,22
Q15303	HER4	0,32	0,82	1,15	1,04	0,38	0,38	0,03	19,13
Q16620	TRKB	0,18	0,72	0,90	1,03	0,37	0,39	0,03	12,56
P00533	EGFR	0,18	0,84	1,02	1,02	0,38	0,38	0,01	29,89
P51451	BLK	0,30	0,82	1,12	1,02	0,40	0,39	0,02	15,56
Q14289	FAK2	0,24	0,81	1,05	1,01	0,39	0,38	0,02	23,78
Q13882	Brk	0,27	0,85	1,12	1,01	0,39	0,39	0,01	40,22
Q08881	ITK	0,15	0,85	1,01	0,99	0,37	0,38	0,02	21,56
P54764	EphA4	0,19	0,71	0,89	0,96	0,36	0,39	0,03	3,33
P07948	Lyn	0,18	0,71	0,90	0,95	0,38	0,38	0,02	14,44
P00519	Abl	0,14	0,84	0,98	0,94	0,38	0,38	0,01	36,11
P29317	EphA2	0,13	0,78	0,91	0,92	0,34	0,34	0,03	4,67
P21860	HER3	0,10	0,73	0,84	0,89	0,36	0,38	0,02	23,44
P08069	IGF1R	0,15	0,71	0,85	0,87	0,36	0,37	0,01	9,22
Q06187	BTK	0,11	0,77	0,88	0,87	0,36	0,35	0,01	12,00
P29376	LTK	0,11	0,72	0,83	0,86	0,35	0,36	0,02	10,25
P42679	CTK	0,09	0,79	0,87	0,85	0,36	0,36	0,01	21,67
P06239	Lck	0,11	0,74	0,85	0,82	0,37	0,37	0,01	18,44
P35968	KDR	0,08	0,71	0,79	0,82	0,35	0,34	0,02	12,22
Q16288	TRKC	0,10	0,72	0,81	0,80	0,35	0,35	0,01	12,11
P07333	FmS/CSFR	0,12	0,66	0,77	0,78	0,34	0,33	0,02	4,78
P07947	Yes	0,08	0,72	0,79	0,77	0,35	0,35	0,02	16,22
P09769	Fgr	0,12	0,62	0,74	0,77	0,33	0,32	0,03	5,67
P06213	InSR	0,05	0,73	0,77	0,75	0,34	0,34	0,01	14,56
H0Y8A4	RYK	0,12	0,63	0,75	0,73	0,34	0,33	0,02	4,56
Q13308	CCK4/PTK7	0,12	0,60	0,73	0,71	0,34	0,34	0,00	3,00

O60674	JAK2	0,03	0,67	0,70	0,71	0,33	0,33	0,02	15,22
P54756	EphA5	0,09	0,59	0,68	0,68	0,32	0,32	NA	3,00
P29320	EphA3	0,09	0,56	0,65	0,65	0,32	0,32	0,00	3,00
P06241	Fyn	0,05	0,64	0,69	0,64	0,32	0,30	0,03	10,11
P14616	IRR	0,03	0,50	0,53	0,53	0,25	0,25	0,01	3,60

Table S4

Kinase Uniprot ID	Kinase Name	Mean Specificity Score (Qsp)	Mean Significance Score (Qsg)	Mean Final Score (Qmean)	Median Final score (Q)	Mean Kinase Statistic (smean)	Median Kinase Statistic (s)	SD Kinase Statitistic (SD)	Mean peptide set size
Q13237	PKG2	2,61	1,42	4,03	4,08	-0,42	-0,42	0,02	39,44
O15111	IKK[alpha]	2,06	1,72	3,78	4,00	-0,71	-0,72	0,01	3,67
Q13976	PKG1	2,65	1,22	3,87	3,92	-0,38	-0,39	0,01	42,22
Q16566	CaMK4	2,43	1,52	3,94	3,92	-0,56	-0,52	0,07	12,44
P17612	PKA[alpha]	2,67	1,19	3,86	3,88	-0,37	-0,37	0,01	55,56
Q9UBS0	p70S6K[beta]	2,27	1,22	3,50	3,67	-0,43	-0,43	0,07	25,89
P51817	PRKX	1,99	1,13	3,12	3,40	-0,35	-0,34	0,02	34,67
P17252	PKC[alpha]	1,73	1,14	2,87	2,92	-0,36	-0,37	0,02	30,22
P68400	CK2[alpha]1	0,92	1,55	2,48	2,51	-0,51	-0,51	0,00	4,00
Q05655	PKC[delta]	1,33	1,10	2,43	2,38	-0,35	-0,34	0,02	23,44
P31749	Akt1/PKB[alpha]	1,28	1,08	2,36	2,34	-0,33	-0,34	0,03	30,11
Q15349	RSK1/p90RSK	0,82	1,36	2,18	2,20	-0,41	-0,41	0,00	6,00
O94921	PFTAIRE1	0,91	1,39	2,30	2,19	-0,51	-0,48	0,07	3,86
Q13131	AMPK[alpha]1	1,07	1,22	2,29	2,19	-0,39	-0,38	0,04	11,78
Q15139	PKD1	0,75	1,27	2,02	2,03	-0,40	-0,40	0,00	6,00
Q96Q40	PFTAIRE2	0,61	1,21	1,81	1,97	-0,34	-0,35	0,05	6,78
Q04759	PKC[theta]	0,81	0,94	1,74	1,90	-0,30	-0,32	0,03	22,33
Q02156	PKC[epsilon]	0,80	0,90	1,70	1,88	-0,29	-0,32	0,06	20,00
O96017	CHK2	0,96	0,94	1,90	1,79	-0,31	-0,31	0,05	20,78
P49137	MAPKAPK2	0,92	0,95	1,87	1,78	-0,29	-0,29	0,03	30,44
Q9UQM7	CaMK2[alpha]	0,56	1,19	1,75	1,74	-0,41	-0,41	0,00	3,00
Q13627	DYRK1A	0,60	1,10	1,71	1,71	0,43	0,43	0,00	3,00
P31751	Akt2/PKB[beta]	0,76	0,89	1,66	1,70	-0,28	-0,28	0,03	28,11
O14920	IKK[beta]	0,48	1,09	1,57	1,66	-0,34	-0,37	0,04	3,67
Q16644	MAPKAPK3	0,74	0,85	1,59	1,64	-0,26	-0,26	0,02	35,22
P24723	PKC[eta]	0,58	0,72	1,30	1,63	-0,23	-0,30	0,13	15,00
O75676	MSK2	0,52	1,11	1,63	1,63	-0,39	-0,39	0,00	3,00
P11309	Pim1	0,82	0,78	1,60	1,62	-0,24	-0,24	0,01	64,78
Q13535	ATR	0,53	1,06	1,59	1,58	-0,29	-0,29	0,00	9,89
Q9P1W9	Pim2	0,63	0,80	1,43	1,47	-0,25	-0,25	0,02	40,56
Q96L96	AlphaK1	0,39	0,89	1,28	1,44	0,30	0,33	0,06	3,67
Q15759	p38[beta]	0,40	0,93	1,33	1,39	0,27	0,27	0,03	6,83
Q9HBY8	SGK2	0,51	0,84	1,35	1,38	-0,26	-0,26	0,04	17,56
P27361	ERK1	0,35	0,75	1,09	1,25	0,21	0,23	0,05	23,22
Q86V86	Pim3	0,56	0,73	1,29	1,24	-0,22	-0,22	0,01	60,44
Q15131	CDK10	0,38	0,82	1,20	1,19	-0,26	-0,27	0,07	6,11
O14965	AurA/Aur2	0,45	0,78	1,23	1,18	-0,30	-0,29	0,02	5,11
P05129	PKC[gamma]	0,36	0,64	1,00	1,18	-0,21	-0,24	0,08	15,22
O75116	ROCK2	0,39	0,86	1,25	1,15	-0,25	-0,23	0,11	4,80
Q13164	ERK5	0,37	0,64	1,01	1,11	0,22	0,24	0,07	13,78
P49840	GSK3[alpha]	0,26	0,80	1,06	1,05	0,21	0,21	0,02	4,67
O14757	CHK1	0,38	0,67	1,05	1,05	-0,24	-0,24	0,04	9,00
P28482	ERK2	0,33	0,71	1,04	1,05	0,21	0,21	0,03	22,00
P53778	p38[gamma]	0,29	0,57	0,86	0,97	0,20	0,22	0,05	10,67
O43930	PRKY	0,29	0,54	0,83	0,94	-0,19	-0,21	0,13	12,33
P41279	COT	0,21	0,73	0,94	0,94	0,18	0,18	0,00	5,00
P06493	CDC2/CDK1	0,25	0,63	0,88	0,86	0,19	0,18	0,01	17,00
O43293	DAPK3	0,20	0,55	0,75	0,81	-0,19	-0,21	0,04	3,11
Q9BWU1	CDK11	0,26	0,54	0,80	0,81	0,21	0,21	0,00	5,00
P16066	ANP[alpha]	0,24	0,55	0,78	0,77	-0,18	-0,19	0,04	25,00
Q96GD4	AurB/Aur1	0,26	0,60	0,86	0,77	-0,20	-0,18	0,05	5,11
Q15418	RSK3	0,25	0,51	0,75	0,76	-0,18	-0,18	0,00	10,00
Q16512	PKN1/PRK1	0,17	0,39	0,56	0,74	0,10	0,19	0,13	6,00
P41743	PKC[iota]	0,21	0,46	0,67	0,73	-0,17	-0,18	0,04	15,22
O75582	MSK1	0,23	0,50	0,73	0,73	-0,18	-0,18	0,00	8,00
O15075	DCAMKL1	0,24	0,48	0,71	0,71	-0,21	-0,21	0,00	4,00
Q14164	IKK[epsilon]	0,34	0,60	0,94	0,69	-0,21	-0,16	0,15	7,56
P49674	CK1[epsilon]	0,16	0,42	0,58	0,68	-0,13	-0,15	0,06	4,89
Q96S38	RSKL1	0,16	0,41	0,58	0,58	-0,17	-0,17	0,00	3,00
O00141	SGK1	0,14	0,42	0,56	0,57	0,15	0,15	0,00	3,00
O76039	CDKL5	0,29	0,53	0,82	0,55	0,22	0,15	0,13	6,25
Q8IWB6	SgK307	0,10	0,27	0,37	0,50	0,10	0,14	0,08	4,11
P51812	RSK2	0,13	0,35	0,48	0,48	-0,11	-0,11	0,00	9,00
P23443	p70S6K	0,19	0,39	0,58	0,46	-0,14	-0,12	0,07	19,67
Q9UPZ9	ICK	0,18	0,36	0,55	0,46	0,17	0,16	0,01	3,33
Q05513	PKC[zeta]	0,17	0,29	0,46	0,45	-0,14	-0,14	0,01	9,56
P48729	CK1[alpha]	0,08	0,31	0,40	0,44	-0,08	-0,10	0,06	13,89
Q00534	CDK6	0,15	0,34	0,48	0,44	0,13	0,11	0,03	6,89
P50750	CDK9	0,10	0,26	0,36	0,43	0,09	0,12	0,03	9,67
P42345	mTOR/FRAP	0,12	0,28	0,40	0,43	-0,11	-0,12	0,04	4,89
P24941	CDK2	0,11	0,36	0,47	0,43	0,12	0,12	0,03	18,89
P11802	CDK4	0,10	0,24	0,35	0,38	0,09	0,10	0,03	8,22
P10398	ARAF	0,12	0,24	0,36	0,36	-0,13	-0,13	0,00	3,00
P15056	BRAF	0,10	0,22	0,32	0,36	-0,11	-0,13	0,05	3,33
P53355	DAPK1	0,12	0,24	0,36	0,36	-0,12	-0,12	0,00	3,00
P49841	GSK3[beta]	0,10	0,25	0,35	0,34	0,09	0,09	0,00	5,89
Q92772	CDKL2	0,12	0,22	0,34	0,33	0,13	0,13	0,00	3,33
Q9Y6S9	RSKL2	0,10	0,23	0,33	0,32	-0,02	-0,02	0,10	3,50
Q00532	CDKL1	0,14	0,30	0,44	0,32	0,12	0,09	0,11	6,00
Q8TD08	ERK7	0,10	0,21	0,31	0,31	0,09	0,09	NA	4,00
Q00537	PCTAIRE2	0,07	0,19	0,27	0,30	0,08	0,10	0,03	3,20
Q9UIK4	DAPK2	0,09	0,31	0,40	0,28	0,01	-0,06	0,10	5,13

Q00535	CDK5	0,08	0,20	0,28	0,28	0,08	0,08	0,00	10,00
Q13464	ROCK1	0,11	0,29	0,40	0,27	0,08	0,08	0,10	4,71
Q13153	PAK1	0,09	0,17	0,26	0,26	-0,08	-0,08	0,00	6,00
O15264	p38[delta]	0,05	0,21	0,26	0,25	0,07	0,08	0,02	19,22
P48730	CK1[delta]	0,10	0,27	0,37	0,25	-0,06	-0,06	0,09	4,11
P45984	JNK2	0,05	0,23	0,29	0,23	0,08	0,07	0,02	21,56
O95819	HGK/ZC1	0,07	0,16	0,23	0,23	-0,07	-0,07	0,04	5,71
Q9UKE5	TNIK/ZC2	0,06	0,13	0,19	0,19	-0,07	-0,07	0,00	3,00
P05771	PKC[beta]	0,04	0,10	0,14	0,14	-0,05	-0,05	0,00	10,00
Q9UHD2	TBK1	0,08	0,20	0,28	0,14	0,06	0,04	0,06	5,67
P04049	RAF1	0,05	0,09	0,15	0,14	-0,01	-0,04	0,05	6,33
P45983	JNK1	0,01	0,12	0,13	0,13	0,05	0,05	0,01	28,44
P53779	JNK3	0,02	0,13	0,15	0,13	0,05	0,05	0,01	27,89
Q00526	CDK3	0,03	0,10	0,13	0,12	-0,03	-0,03	0,03	13,56
Q8NI60	ADCK3	0,13	0,30	0,43	0,12	-0,10	-0,03	0,10	9,56
O60285	NuaK1	0,14	0,34	0,48	0,11	-0,08	0,02	0,18	5,17
P50613	CDK7	0,03	0,10	0,13	0,09	0,02	0,03	0,04	14,33
Q16539	MAPK14	0,02	0,09	0,12	0,07	-0,01	0,01	0,04	15,33

Table S5

PKCε

Sample	PKCε Area	Vinculin Area	PKCε/Vinculin Area	Mean	PKCε/Vinculin Norm. to UT-10	Mean
ET-01	23222,62	20743,48	1,12		1,04	
ET-03	24993,97	24540,72	1,02		0,95	
ET-04	27606,74	24359,87	1,13		1,06	
ET-05	30216,11	22732,48	1,33	1,22	1,24	1,14
ET-06	27834,45	21600,41	1,29		1,20	
ET-09	28241,52	23096,07	1,22		1,14	
ET-11	36128,66	25251,41	1,43		1,33	
UT-01	35149,91	27619,26	1,27		1,19	
UT-04	31212,30	20861,80	1,50		1,40	
UT-06	19621,23	16930,68	1,16		1,08	
UT-08	28540,16	23112,05	1,23	1,08	1,15	1,00
UT-10	25143,84	23450,75	1,07		1,00	
UT-13	21486,21	29473,99	0,73		0,68	
UT-14	16976,48	29386,50	0,58		0,54	

p38-MAPKδ

Sample	p38-MAPKδ Area	Vinculin Area	p38-MAPKδ/Vinculin Area	Mean	p38-MAPKδ/Vinculin Norm. to UT-08	Mean
ET-01	10980,27	20743,48	0,53		1,09	
ET-03	15476,05	24540,72	0,63		1,29	
ET-04	16437,70	24359,87	0,67		1,38	
ET-05	16172,63	22732,48	0,71	0,62	1,46	1,28
ET-06	12775,63	21600,41	0,59		1,21	
ET-09	14360,87	23096,07	0,62		1,28	
ET-11	15388,53	25251,41	0,61		1,25	
UT-01	14586,29	27619,26	0,53		1,08	
UT-04	12380,34	20861,80	0,59		1,22	
UT-06	12082,41	16930,68	0,71		1,46	
UT-08	11266,39	23112,05	0,49	0,46	1,00	0,94
UT-10	8967,44	23450,75	0,38		0,78	
UT-13	10026,02	29473,99	0,34		0,70	
UT-14	4307,52	29386,50	0,15		0,30	

ZAP70

Sample	ZAP70 Area	Vinculin Area	ZAP70/Vinculin Area	Mean	ZAP70/Vinculin Norm. to UT-06	Mean
ET-01	26756,10	20743,48	1,29		1,23	
ET-03	28158,56	24540,72	1,15		1,09	
ET-04	32815,18	24359,87	1,35		1,28	
ET-05	32035,15	22732,48	1,41	1,12	1,34	1,06
ET-06	20939,25	21600,41	0,97		0,92	
ET-09	18477,47	23096,07	0,80		0,76	
ET-11	22257,13	25251,41	0,88		0,84	
UT-01	27622,54	27619,26	1,00		0,95	
UT-04	16844,16	20861,80	0,81		0,77	
UT-06	17823,97	16930,68	1,05		1,00	
UT-08	30042,79	23112,05	1,30	1,10	1,23	1,04
UT-10	34261,42	23450,75	1,46		1,39	
UT-13	34477,42	29473,99	1,17		1,11	
UT-14	26120,82	29386,50	0,89		0,84	

FGFR2

Sample	FGFR2 Area	Vinculin Area	FGFR2/Vinculin Area	Mean	FGFR2/Vinculin Norm. to UT-01	Mean
ET-01						
ET-03	19954300,00	24540,72	813,11		1,25	
ET-04	18580735,00	24359,87	762,76		1,17	
ET-05	16125664,00	22732,48	709,37	613,78	1,09	0,94
ET-06	10659744,00	21600,41	493,50		0,76	
ET-09	8096359,00	23096,07	350,55		0,54	
ET-11	13973643,00	25251,41	553,38		0,85	
UT-01	17942664,00	27619,26	649,64		1,00	
UT-04	15334371,00	20861,80	735,05		1,13	
UT-06	11244380,00	16930,68	664,14		1,02	
UT-08	12554765,00	23112,05	543,21	613,76	0,84	0,94
UT-10	17878907,00	23450,75	762,40		1,17	
UT-13	11088815,00	29473,99	376,22		0,58	
UT-14	16621836,00	29386,50	565,63		0,87	

Table S6

Biological Process (GO)

#term ID	term description	observed gene count	background gene count	false discovery rate	matching proteins in your network (labels)
GO:0006468	protein phosphorylation	15,00	923,00	0,0000000	CAMK4,CHUK,CSNK2A1,FGFR1,FGFR2,FGFR3,FGFR4,PRKACA,PRKCA,PRKCD,PRKG1,PRKG2,PRKX,RPS6KB2,ZAP7
GO:0018193	peptidyl-amino acid modification	13,00	842,00	0,0000000	CAMK4,CHUK,CSNK2A1,FGFR1,FGFR2,FGFR3,FGFR4,PRKACA,PRKCA,PRKCD,PRKG2,PRKX,ZAP70
GO:0046777	protein autophosphorylation	9,00	198,00	0,0000000	CAMK4,FGFR1,FGFR2,FGFR3,FGFR4,PRKACA,PRKG2,PRKX,ZAP70
GO:0018105	peptidyl-serine phosphorylation	8,00	173,00	0,0000000	CAMK4,CHUK,CSNK2A1,PRKACA,PRKCA,PRKCD,PRKG2,PRKX
GO:0035556	intracellular signal transduction	12,00	1528,00	0,0000000	CAMK4,CHUK,FGFR1,FGFR2,FGFR3,FGFR4,PRKACA,PRKCA,PRKCD,PRKG1,RPS6KB2,ZAP70
GO:0018108	peptidyl-tyrosine phosphorylation	6,00	195,00	0,0000005	FGFR1,FGFR2,FGFR3,FGFR4,PRKCD,ZAP70
GO:0007165	signal transduction	14,00	4738,00	0,0000023	CAMK4,CHUK,CSNK2A1,FGFR1,FGFR2,FGFR3,FGFR4,PRKACA,PRKCA,PRKCD,PRKG1,PRKG2,RPS6KB2,ZAP70
GO:0036092	phosphatidylinositol-3-phosphate biosynthetic process	4,00	51,00	0,0000053	FGFR1,FGFR2,FGFR3,FGFR4
GO:1902531	regulation of intracellular signal transduction	10,00	1764,00	0,0000053	CHUK,CSNK2A1,FGFR1,FGFR2,FGFR3,FGFR4,PRKACA,PRKCA,PRKCD,ZAP70
GO:0009967	positive regulation of signal transduction	9,00	1493,00	0,0000181	CHUK,CSNK2A1,FGFR1,FGFR2,FGFR3,FGFR4,PRKCA,PRKCD,ZAP70
GO:0048584	positive regulation of response to stimulus	10,00	2054,00	0,0000181	CHUK,CSNK2A1,FGFR1,FGFR2,FGFR3,FGFR4,PRKACA,PRKCA,PRKCD,ZAP70
GO:0008543	fibroblast growth factor receptor signaling pathway	4,00	80,00	0,0000233	FGFR1,FGFR2,FGFR3,FGFR4
GO:0018107	peptidyl-threonine phosphorylation	4,00	80,00	0,0000233	CSNK2A1,PRKACA,PRKCA,PRKCD
GO:0090330	regulation of platelet aggregation	3,00	17,00	0,0000233	PRKCA,PRKCD,PRKG1
GO:0051247	positive regulation of protein metabolic process	9,00	1587,00	0,0000251	CSNK2A1,FGFR1,FGFR2,FGFR3,FGFR4,PRKACA,PRKCA,PRKCD,RPS6KB2
GO:0007166	cell surface receptor signaling pathway	10,00	2198,00	0,0000272	CHUK,CSNK2A1,FGFR1,FGFR2,FGFR3,FGFR4,PRKACA,PRKCA,PRKCD,ZAP70
GO:0051173	positive regulation of nitrogen compound metabolic process	11,00	2946,00	0,0000292	CAMK4,CHUK,CSNK2A1,FGFR1,FGFR2,FGFR3,FGFR4,PRKACA,PRKCA,PRKCD,RPS6KB2
GO:0007169	transmembrane receptor protein tyrosine kinase signaling pathway	6,00	499,00	0,0000438	FGFR1,FGFR2,FGFR3,FGFR4,PRKCA,ZAP70
GO:0010604	positive regulation of macromolecule metabolic process	11,00	3081,00	0,0000438	CAMK4,CHUK,CSNK2A1,FGFR1,FGFR2,FGFR3,FGFR4,PRKACA,PRKCA,PRKCD,RPS6KB2
GO:0046854	phosphatidylinositol phosphorylation	4,00	103,00	0,0000438	FGFR1,FGFR2,FGFR3,FGFR4

Table S7

#term ID	KEGG pathway term description	observed gene count	background gene count	false discovery rate	matching proteins in your network
hsa04014	Ras signaling pathway	8,00	228,00	0,0000000	CHUK,FGFR1,FGFR2,FGFR3,FGFR4,PRKACA,PRKCA,ZAP70
hsa04010	MAPK signaling pathway	7,00	293,00	0,0000001	CHUK,FGFR1,FGFR2,FGFR3,FGFR4,PRKACA,PRKCA
hsa05200	Pathways in cancer	8,00	515,00	0,0000001	CHUK,FGFR1,FGFR2,FGFR3,FGFR4,PRKACA,PRKCA,RPS6KB2
hsa04151	PI3K-Akt signaling pathway	7,00	348,00	0,0000001	CHUK,FGFR1,FGFR2,FGFR3,FGFR4,PRKCA,RPS6KB2
hsa01521	EGFR tyrosine kinase inhibitor resistance	4,00	78,00	0,0000103	FGFR2,FGFR3,PRKCA,RPS6KB2
hsa04015	Rap1 signaling pathway	5,00	203,00	0,0000103	FGFR1,FGFR2,FGFR3,FGFR4,PRKCA
hsa04540	Gap junction	4,00	87,00	0,0000114	PRKACA,PRKCA,PRKG1,PRKG2
hsa04713	Circadian entrainment	4,00	93,00	0,0000114	PRKACA,PRKCA,PRKG1,PRKG2
hsa04714	Thermogenesis	5,00	228,00	0,0000114	FGFR1,PRKACA,PRKG1,PRKG2,RPS6KB2
hsa04970	Salivary secretion	4,00	86,00	0,0000114	PRKACA,PRKCA,PRKG1,PRKG2
hsa04270	Vascular smooth muscle contraction	4,00	119,00	0,0000253	PRKACA,PRKCA,PRKCD,PRKG1
hsa04550	Signaling pathways regulating pluripotency of stem cells	4,00	138,00	0,0000411	FGFR1,FGFR2,FGFR3,FGFR4
hsa04923	Regulation of lipolysis in adipocytes	3,00	53,00	0,0001100	PRKACA,PRKG1,PRKG2
hsa05205	Proteoglycans in cancer	4,00	195,00	0,0001300	FGFR1,PRKACA,PRKCA,RPS6KB2
hsa04720	Long-term potentiation	3,00	64,00	0,0001400	CAMK4,PRKACA,PRKCA
hsa04730	Long-term depression	3,00	60,00	0,0001400	PRKCA,PRKG1,PRKG2
hsa04810	Regulation of actin cytoskeleton	4,00	205,00	0,0001400	FGFR1,FGFR2,FGFR3,FGFR4
hsa05031	Amphetamine addiction	3,00	65,00	0,0001400	CAMK4,PRKACA,PRKCA
hsa05230	Central carbon metabolism in cancer	3,00	65,00	0,0001400	FGFR1,FGFR2,FGFR3
hsa04064	NF-kappa B signaling pathway	3,00	93,00	0,0003100	CHUK,CSNK2A1,ZAP70

Table S8

#term ID	Reactome pathway<br>term description	observed gene count	background gene count	false discovery rate	matching proteins in your network
HSA-9006925	Intracellular signaling by second messengers	9,00	274,00	0,00000000003	CAMK4,CHUK,CSNK2A1,FGFR4,PRKACA,PRKCA,PRKCD,PRKX,RPS6KB2
HSA-111933	Calmodulin induced events	5,00	27,00	0,00000000338	CAMK4,PRKACA,PRKCA,PRKCD,PRKX
HSA-1226099	Signaling by FGFR in disease	4,00	58,00	0,00000365000	FGFR1,FGFR2,FGFR3,FGFR4
HSA-162582	Signal Transduction	11,00	2605,00	0,00000482000	CAMK4,CHUK,CSNK2A1,FGFR4,PRKACA,PRKCA,PRKCD,PRKG1,PRKG2,PRKX,RPS6KB2
HSA-5663202	Diseases of signal transduction	6,00	360,00	0,00000482000	CHUK,FGFR1,FGFR2,FGFR3,FGFR4,RPS6KB2
HSA-1643685	Disease	8,00	1018,00	0,00000578000	CAMK4,CHUK,FGFR1,FGFR2,FGFR3,FGFR4,PRKACA,RPS6KB2
HSA-438064	Post NMDA receptor activation events	3,00	39,00	0,00008550000	CAMK4,PRKACA,PRKX
HSA-112314	Neurotransmitter receptors and postsynaptic signal transmission	4,00	150,00	0,00009090000	CAMK4,PRKACA,PRKCA,PRKX
HSA-9022535	Loss of phosphorylation of MECP2 at T308	2,00	3,00	0,00009780000	CAMK4,PRKACA
HSA-4086398	Ca2+ pathway	3,00	59,00	0,00020000000	PRKCA,PRKG1,PRKG2
HSA-442720	CREB phosphorylation through the activation of Adenylate Cyclase	2,00	8,00	0,00033000000	PRKACA,PRKX
HSA-450520	HuR (ELAVL1) binds and stabilizes mRNA	2,00	8,00	0,00033000000	PRKCA,PRKCD
HSA-9010642	ROBO receptors bind AKAP5	2,00	8,00	0,00033000000	PRKACA,PRKCA
HSA-1257604	PIP3 activates AKT signaling	4,00	242,00	0,00035000000	CHUK,CSNK2A1,FGFR4,RPS6KB2
HSA-2219528	PI3K/AKT Signaling in Cancer	3,00	85,00	0,00043000000	CHUK,FGFR4,RPS6KB2
HSA-4420097	VEGFA-VEGFR2 Pathway	3,00	95,00	0,00053000000	PRKACA,PRKCA,PRKCD
HSA-195721	Signaling by WNT	4,00	293,00	0,00060000000	CSNK2A1,PRKCA,PRKG1,PRKG2
HSA-5218921	VEGFR2 mediated cell proliferation	2,00	17,00	0,00084000000	PRKCA,PRKCD
HSA-111931	PKA-mediated phosphorylation of CREB	2,00	19,00	0,00100000000	PRKACA,PRKX
HSA-1250196	SHC1 events in ERBB2 signaling	2,00	19,00	0,00100000000	PRKCA,PRKCD

Table S9

#term ID	Cellular component term description	observed gene count	background gene count	false discovery rate	matching proteins in your network
GO:0005886	plasma membrane	12,00	5159,00	0,004	CHUK,CSNK2A1,FGFR1,FGFR2,FGFR3,FGFR4,PRKACA,PRKCA,PRKCD,PRKG1,PRKG2,ZAP70
GO:0044459	plasma membrane part	9,00	2651,00	0,004	CHUK,FGFR1,FGFR2,FGFR3,FGFR4,PRKACA,PRKCA,PRKG2,ZAP70
GO:0005737	cytoplasm	15,00	11238,00	0,010	CAMK4,CHUK,CSNK2A1,FGFR1,FGFR2,FGFR3,FGFR4,PRKACA,PRKCA,PRKCD,PRKG1,PRKG2,PRKX,RPS6KB2,ZAP70
GO:0044428	nuclear part	10,00	4359,00	0,010	CAMK4,CHUK,CSNK2A1,FGFR1,FGFR2,PRKACA,PRKCA,PRKCD,PRKG2,RPS6KB2
GO:0005634	nucleus	12,00	6892,00	0,014	CAMK4,CHUK,CSNK2A1,FGFR1,FGFR2,FGFR3,PRKACA,PRKCA,PRKCD,PRKG2,PRKX,RPS6KB2
GO:0005829	cytosol	10,00	4958,00	0,021	CAMK4,CHUK,CSNK2A1,FGFR1,PRKACA,PRKCA,PRKCD,PRKG1,PRKG2,ZAP70
GO:0031981	nuclear lumen	9,00	4030,00	0,021	CAMK4,CHUK,CSNK2A1,FGFR1,FGFR2,PRKACA,PRKCA,PRKCD,RPS6KB2
GO:0043231	intracellular membrane-bounded organelle	14,00	10365,00	0,021	CAMK4,CHUK,CSNK2A1,FGFR1,FGFR2,FGFR3,FGFR4,PRKACA,PRKCA,PRKCD,PRKG1,PRKG2,PRKX,RPS6KB2
GO:0043235	receptor complex	3,00	305,00	0,025	CHUK,FGFR1,ZAP70
GO:0005654	nucleoplasm	8,00	3446,00	0,027	CAMK4,CHUK,CSNK2A1,FGFR2,PRKACA,PRKCA,PRKCD,RPS6KB2
GO:1902911	protein kinase complex	2,00	84,00	0,027	CHUK,PRKACA
GO:0044444	cytoplasmic part	13,00	9377,00	0,029	CAMK4,CHUK,CSNK2A1,FGFR1,FGFR2,FGFR3,FGFR4,PRKACA,PRKCA,PRKCD,PRKG1,PRKG2,ZAP70
GO:0031410	cytoplasmic vesicle	6,00	2226,00	0,044	FGFR1,FGFR2,FGFR3,FGFR4,PRKACA,PRKCD
GO:0005887	integral component of plasma membrane	5,00	1564,00	0,045	CHUK,FGFR1,FGFR2,FGFR3,FGFR4
GO:0005576	extracellular region	6,00	2505,00	0,050	FGFR1,FGFR2,FGFR3,FGFR4,PRKACA,PRKCD
GO:0009898	cytoplasmic side of plasma membrane	2,00	170,00	0,050	CHUK,ZAP70
GO:0012505	endomembrane system	8,00	4347,00	0,050	FGFR2,FGFR3,FGFR4,PRKACA,PRKCA,PRKCD,PRKG1,PRKG2
GO:0098796	membrane protein complex	4,00	1047,00	0,050	CHUK,PRKACA,PRKCA,ZAP70
GO:0098797	plasma membrane protein complex	3,00	502,00	0,050	CHUK,PRKCA,ZAP70
GO:0098802	plasma membrane receptor complex	2,00	158,00	0,050	CHUK,ZAP70

Table S10

Kinase (signaling molecule)	Biological role in lymphocytes/lymphocyte subpopulations
	Tyrosine kinases
FGFR	The specific role of FGFRs in human lymphocytes is poorly studied. FGFRs get activated by autophosphorylation after binding to their ligands FGFs, which were actively secreted under conditions related to strenuous endurance exercise such as hypoxia or ischemia [1,2]. Studies indicate that FGF-responsive T cells may play a role in the migration and proliferation of vascular-smooth muscle cells in vascular and inflammatory lesions [3]. The ligation of FGFR1 on T cells co-stimulates T cell receptor-triggered IL-2 production [4].
ZAP70	ZAP-70 plays an essential role in lymphocyte activation by functioning in the initial step of T cell antigen receptor mediated signal transduction. Furthermore, ZAP-70 is involved in the regulation of adhesion, motility and cytokine expression of mature T cells and contributes to the activation and development of primary B cells. The phosphorylation of specific adapter proteins by ZAP70 leads to T cell proliferation, differentiation and the production of lymphokines https://www.uniprot.org/uniprot/P43403. T cells which lack ZAP-70 show defects in T cell activation and down-stream signaling events [5].
	Serine-/Threonine kinases
PKG (PRKG)	PKGs are regulated by cGMP signaling. [6]. Protein targets for PKGs are involved in the regulation of cellular calcium homeostasis https://www.uniprot.org/uniprot/Q13976. Further, this kinase is positively associated with proteasome activities [10]. There is little information of the biological role of PKGs in human lymphocytes which is limited to its regulatory function in selected lymphocyte subpopulations. PKG1 affects the regulation of gene expression, is involved in the inhibition of T cell proliferation and controls the cytokine production of TH2 cells [7,8]. Moreover, the activation of PKG stimulates the down regulation of interleukin 2 signaling in T cell lines [9].
IKKα (CHUK)	This kinase plays a crucial role in the NF-kappa-B signaling pathway which is stimulated by inflammatory cytokines, bacteria, viruses, DNA damage or other cellular stressors The IKK complex regulates genes encoding signaling molecules implicated in B cell survival as well as lymphoid organogenesis https://www.uniprot.org/uniprot/O15111.
CAMK4	CAMK4 is a calcium/calmodulin-dependent multifunctional protein kinase that regulates gene expression by activating several transcription factors in T cells. In CD4 positive memory T-cells, CAMK4 is essential to link TCR signaling to the production of IL2, IFNγ and IL4 through the modulation of the transcription factors MEF2 and CREB https://www.uniprot.org/uniprot/Q16566.<br>Besides, CAMK4 modulates several processes that contribute to the pathology of autoimmune diseases by regulating the production of IL-17 and IL-2 by T cells [11].
PKAα (PRKACA)	The activity of PKAα depends on the cellular level of cAMP [6] . PKAα inhibits the early and late phase of the antigen-induced activation of T and B lymphocytes [12]. Furthermore, it contributes to the regulation of apoptosis of immature T lymphocytes [13].
P70S6Kβ (RPS6SKB2)	Kinases of the P70S6K group affect important lymphocytic functions such as cell growth, differentiation, proliferation and function as a major regulator in glucose metabolism of T cells [14,15].
PRKX	So far, the biological role of PRKX in lymphocytes is almost unknown due to a lack of studies [6,16].
PKCα (PRKCA)	PKCα is activated by calcium and diacylglycerol (https://www.uniprot.org/uniprot/P17252). This kinase is involved in the activation of the IKK complex and NFκB in T lymphocytes as response to TCR activation [17]. Besides PKCα mediates TCR down regulation[18].
CK2α1 (CSNK2A1)	CK2α1 phophorylates acidic proteins such as casein. This kinase regulates several cellular processes such as transcription, apoptosis and cell cycle progression

	https://www.uniprot.org/uniprot/P68400. Furthermore, it represents a crucial regulator of the balance of Th17 and Treg cell differentiation [19].
PKCδ (PRKCD)	PKCδ is activated by diacylglycerol (https://www.uniprot.org/uniprot/Q05655). This isoform of protein kinase C is involved in the regulation of mitochondrial-dependent apoptosis via phosphorylation of histones at the apoptotic histone residues of T-Cells [20,21]. In addition, PKCδ plays a functional role in the regulation of apoptosis in resting B cells and suppresses the proliferation of B cells [22].

1. Blick, C. *et al.* Hypoxia regulates FGFR3 expression via HIF-1α and miR-100 and contributes to cell survival in non-muscle invasive bladder cancer. *British journal of cancer* **109,** 50–59; 10.1038/bjc.2013.240 (2013).
2. House, S. L. *et al.* Endothelial fibroblast growth factor receptor signaling is required for vascular remodeling following cardiac ischemia-reperfusion injury. *American journal of physiology. Heart and circulatory physiology* **310,** H559-71; 10.1152/ajpheart.00758.2015 (2016).
3. Zhao, X. M. *et al.* Costimulation of human CD4+ T cells by fibroblast growth factor-1 (acidic fibroblast growth factor). *Journal of immunology (Baltimore, Md. : 1950)* **155,** 3904–3911 (1995).
4. Kos, F. J. & Chin, C. S. Costimulation of T cell receptor-triggered IL-2 production by Jurkat T cells via fibroblast growth factor receptor 1 upon its engagement by CD56. *Immunology and cell biology* **80,** 364–369; 10.1046/j.1440-1711.2002.01098.x (2002).
5. Kaur, M., Singh, M. & Silakari, O. Insight into the therapeutic aspects of 'Zeta-Chain Associated Protein Kinase 70 kDa' inhibitors. A review. *Cellular signalling* **26,** 2481–2492; 10.1016/j.cellsig.2014.06.017 (2014).
6. Pearce, L. R., Komander, D. & Alessi, D. R. The nuts and bolts of AGC protein kinases. *Nature reviews. Molecular cell biology* **11,** 9–22; 10.1038/nrm2822 (2010).
7. He, B. & Weber, G. F. Phosphorylation of NF-kappaB proteins by cyclic GMP-dependent kinase. A noncanonical pathway to NF-kappaB activation. *European journal of biochemistry* **270,** 2174–2185 (2003).
8. Gomes, B. *et al.* The cGMP/protein kinase G pathway contributes to dihydropyridine-sensitive calcium response and cytokine production in TH2 lymphocytes. *The Journal of biological chemistry* **281,** 12421–12427; 10.1074/jbc.M510653200 (2006).
9. Taffs, R. E. & Sitkovsky, M. V. Modulation of the effector functions of cytolytic T-lymphocytes with synthetic peptide inhibitors of protein kinases. *Journal of pharmaceutical sciences* **81,** 37–44; 10.1002/jps.2600810108 (1992).
10. Zhang, J., Gao, Z., Yin, J., Quon, M. J. & Ye, J. S6K directly phosphorylates IRS-1 on Ser-270 to promote insulin resistance in response to TNF-(alpha) signaling through IKK2. *The Journal of biological chemistry* **283,** 35375–35382; 10.1074/jbc.M806480200 (2008).
11. Ferretti, A. P., Bhargava, R., Dahan, S., Tsokos, M. G. & Tsokos, G. C. Calcium/Calmodulin Kinase IV Controls the Function of Both T Cells and Kidney Resident Cells. *Frontiers in immunology* **9,** 2113; 10.3389/fimmu.2018.02113 (2018).
12. Skålhegg, B. S. *et al.* Protein kinase A (PKA)--a potential target for therapeutic intervention of dysfunctional immune cells. *Current drug targets* **6,** 655–664 (2005).
13. Zambon, A. C., Wilderman, A., Ho, A. & Insel, P. A. Increased expression of the pro-apoptotic protein BIM, a mechanism for cAMP/protein kinase A (PKA)-induced apoptosis of immature T cells. *The Journal of biological chemistry* **286,** 33260–33267; 10.1074/jbc.M111.268979 (2011).
14. Jastrzebski, K., Hannan, K. M., Tchoubrieva, E. B., Hannan, R. D. & Pearson, R. B. Coordinate regulation of ribosome biogenesis and function by the ribosomal protein S6 kinase, a key mediator of mTOR function. *Growth factors (Chur, Switzerland)* **25,** 209–226; 10.1080/08977190701779101 (2007).
15. Palmer, C. S. *et al.* Regulators of Glucose Metabolism in CD4+ and CD8+ T Cells. *International reviews of immunology* **35,** 477–488; 10.3109/08830185.2015.1082178 (2016).
16. Huang, S., Li, Q., Alberts, I. & Li, X. PRKX, a Novel cAMP-Dependent Protein Kinase Member, Plays an Important Role in Development. *Journal of cellular biochemistry* **117,** 566–573; 10.1002/jcb.25304 (2016).

17. Trushin, S. A. *et al.* Protein kinase Calpha (PKCalpha) acts upstream of PKCtheta to activate IkappaB kinase and NF-kappaB in T lymphocytes. *Molecular and cellular biology* **23,** 7068–7081; 10.1128/mcb.23.19.7068-7081.2003 (2003).
18. Essen, M. von *et al.* Protein kinase C (PKC) alpha and PKC theta are the major PKC isotypes involved in TCR down-regulation. *Journal of immunology (Baltimore, Md. : 1950)* **176,** 7502–7510 (2006).
19. Jang, S. W. *et al.* Casein kinase 2 is a critical determinant of the balance of Th17 and Treg cell differentiation. *Experimental & molecular medicine* **49,** e375; 10.1038/emm.2017.132 (2017).
20. Lim, P. S., Sutton, C. R. & Rao, S. Protein kinase C in the immune system. From signalling to chromatin regulation. *Immunology* **146,** 508–522; 10.1111/imm.12510 (2015).
21. Park, C.-H. & Kim, K.-T. Apoptotic phosphorylation of histone H3 on Ser-10 by protein kinase Cδ. *PloS one* **7,** e44307; 10.1371/journal.pone.0044307 (2012).
22. Mecklenbräuker, I., Kalled, S. L., Leitges, M., Mackay, F. & Tarakhovsky, A. Regulation of B-cell survival by BAFF-dependent PKCdelta-mediated nuclear signalling. *Nature* **431,** 456–461; 10.1038/nature02955 (2004).

Figure Legends

Figure S1. Ponceau S staining and segmentation of the membrane. The membrane was reversibly stained with Ponceau S dye for verification of equal sample loading (red colored protein bands) and efficient transfer on the nitrocellulose membrane during western blotting. Blue and orange bands on the left indicate the different sizes of marker proteins with a specific molecular weight (S1A). The PageRuler Prestained Protein Ladder from Thermo Scientific was used as size standard. The membrane was cut into an upper and a lower section according to the scheme depicted on the right (S1B) prior incubation with the different antibodies of interest.

Figure S2: Full-length blot of protein kinase C epsilon (PKCε).

Figure S3: Full-length blot of zeta- chain-associated protein kinase (ZAP70).

Figure S4. Full-length blot of fibroblast growth factor receptor 2 (FGFR2).

Figure S5. Full-length blot of Vinculin (loading control).

Figure S6. Full-length blot of p38-delta mitogen-activated protein kinase (p38δ).

Fig. S1

Fig. 1A

Ponceau S staining

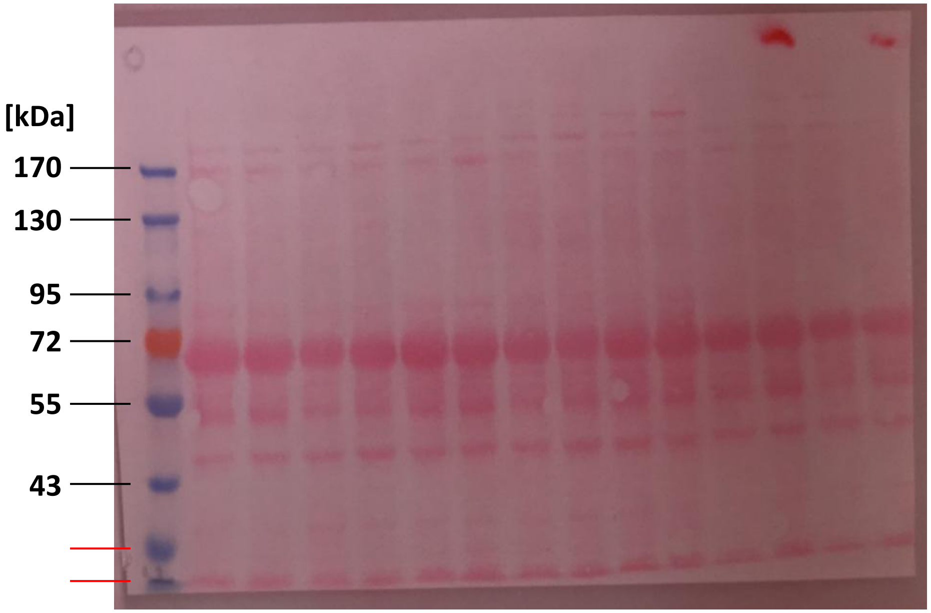

Fig. 1B

PageRuler Prestained Protein ladder

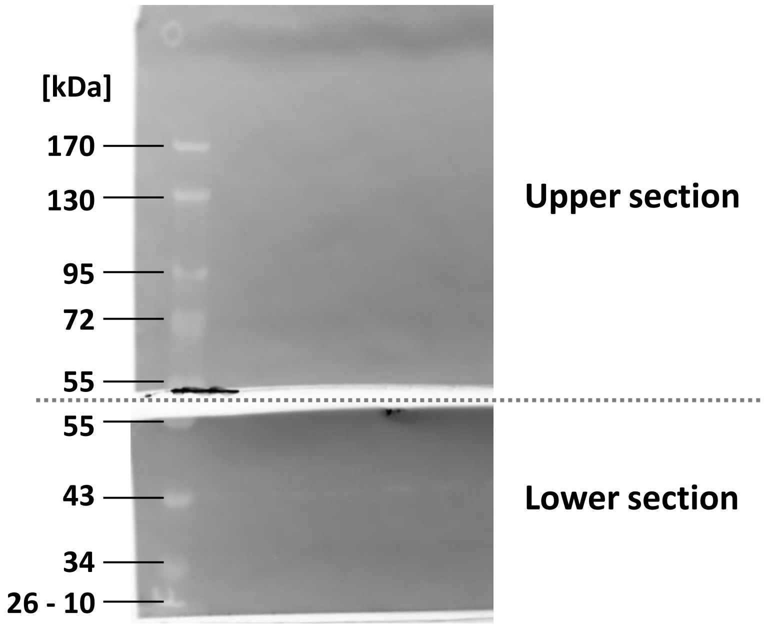

The membrane was cut after protein transfer at 55 kDa.

Fig. S2

PKCε

Section: upper part of the membrane
Order: antibody #1
Molecular weight: 82 kDa (prediction)
Dilution: 1/1000 in milk
Manufacturer: Cell signaling
ID: #2683
Species: Rabbit
Exposure time: 1 min
Substrate: ECL Prime (sensitivity: medium)

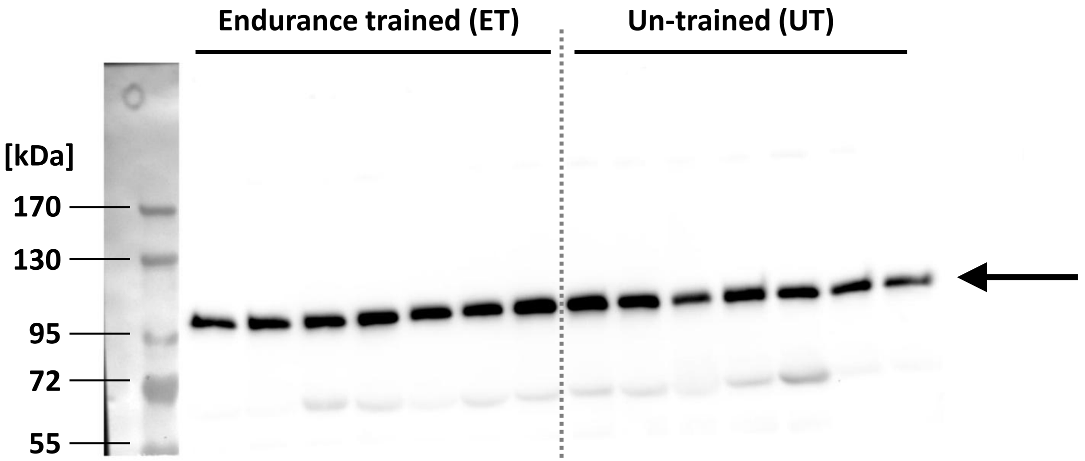

Fig. S3

ZAP-70

Section: upper part of the membrane
Order: antibody #2
Molecular weight: 70 kDA (prediction)
Dilution: 1/500 in BSA
Manufacturer: Cell signaling
ID: #3165
Species: Rabbit
Exposure time: 6 sec
Substrate: Luminata (sensitivity: low)

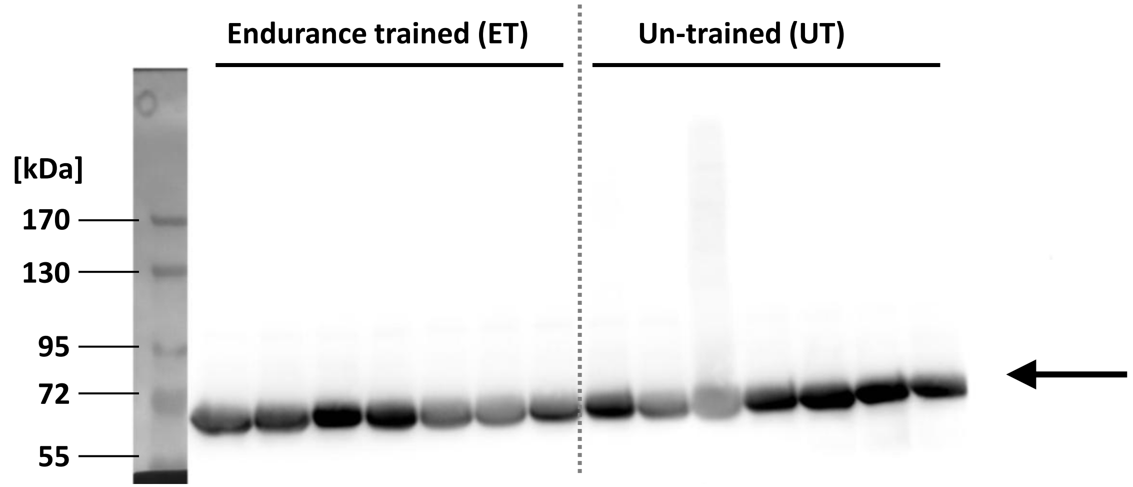

Fig. S4

FGFR2

Section: upper part of the membrane
Order: antibody #3
Molecular weight: 110 kDa (prediction), most antibodies detect the FGFR2 protein at 140 kDa
Dilution: 1/1000 in milk
Manufacturer: Abcam
ID: ab10648
Species: Rabbit
Exposure time: 2 min
Substrate: Femto (sensitivity: high)

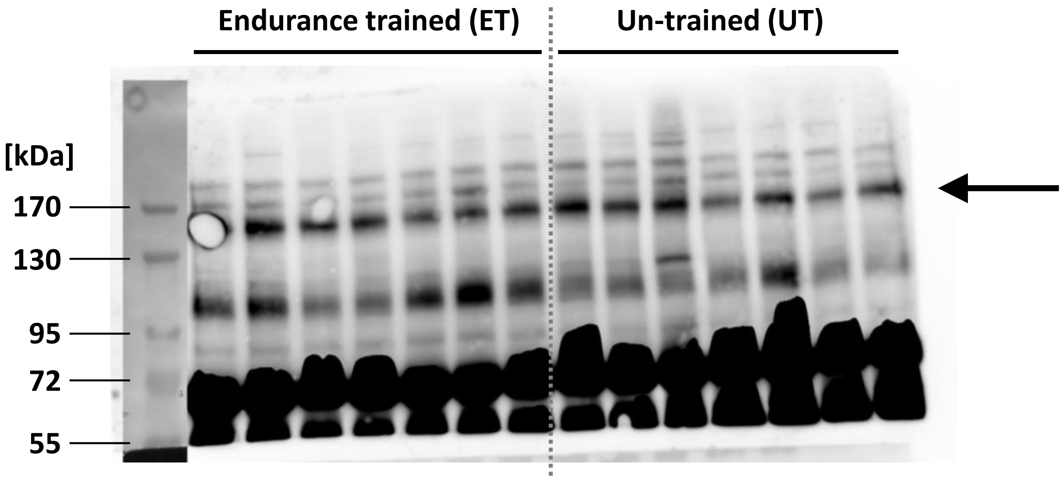

Fig. S5

Vinculin

Section: upper part of the membrane
Order: antibody #4
Molecular weight: 130 kDa
Dilution: 1/2000 in milk
Manufacturer: Abcam
ID: ab18058
Species: Mouse
Exposure time: 1 min
Substrate: Luminata (sensitivity: low)

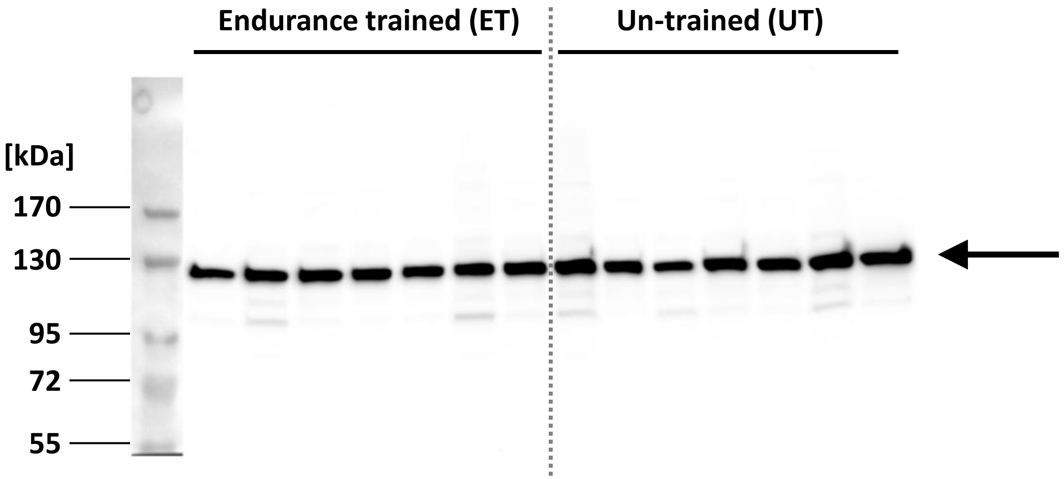

Fig. S6

P38-δ MAPK

Section: lower part of the membrane
Order: antibody #1
Molecular weight: 43 kDa
Dilution: 1/500 in BSA
Manufacturer: Cell signaling
ID: #2308
Species: Rabbit
Exposure time: 30 sec
Substrate: Luminata (sensitivity : low)

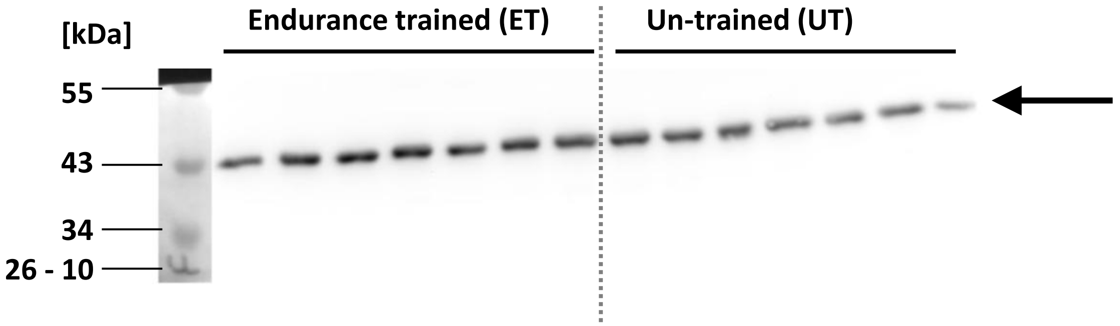

Supplement: Supplementary file 1 — Supplementary information. [file 41598_2020_57676_MOESM1_ESM.pdf]
